# Risk1, a Phosphatidylinositol 3-Kinase Effector, Promotes Rickettsia typhi Intracellular Survival

**DOI:** 10.1128/mBio.00820-20

**Published:** 2020-06-16

**Authors:** Oliver H. Voss, Joseph J. Gillespie, Stephanie S. Lehman, Sherri A. Rennoll, Magda Beier-Sexton, M. Sayeedur Rahman, Abdu F. Azad

**Affiliations:** a Department of Microbiology and Immunology, University of Maryland School of Medicine, Baltimore, Maryland, USA; Ohio State University

**Keywords:** *R. typhi*, Risk1, Rab5, EEA1, ubiquitin, Beclin-1, LC3b, phagosome escape, endosomal trafficking, phosphoinositide metabolism, autophagosomal maturation, bacterial escape

## Abstract

To establish a habitable intracellular niche, various pathogenic bacteria secrete effectors that target intracellular trafficking and modulate phosphoinositide (PI) metabolism. Murine typhus, caused by the obligate intracellular bacterium Rickettsia typhi, remains a severe disease in humans. However, the mechanisms by which R. typhi effector molecules contribute to internalization by induced phagocytosis and subsequent phagosomal escape into the cytosol to facilitate the intracellular growth of the bacteria remain ill-defined. Here, we characterize a new molecule, Risk1, as a phosphatidylinositol 3-kinase (PI3K) secreted effector and the first bacterial secretory kinase with both class I and III PI3K activities. Inactivation of Risk1 PI3K activities reduced the phosphorylation of phosphatidylinositol 4,5-bisphosphate to phosphatidylinositol 3,4,5-trisphosphate within the host, which consequently diminished host colonization by R. typhi. During infection, Risk1 targets the Rab5-EEA1-phosphatidylinositol 3-phosphate [PI(3)P] signaling axis to promote bacterial phagosomal escape. Subsequently, R. typhi undergoes ubiquitination and induces host autophagy; however, maturation to autolysosomes is subverted to support intracellular growth. Intriguingly, only enzymatically active Risk1 binds the Beclin-1 core complex and contributes to R. typhi-induced autophagosome formation. In sum, our data suggest that Risk1, with dual class I and class III PI3K activities, alters host PI metabolism and consequently subverts intracellular trafficking to facilitate intracellular growth of R. typhi.

## Abstract (precis)

*Rickettsia* species are Gram-negative obligate intracellular bacteria that infect a wide range of eukaryotes and vertebrates. In particular, human body louse-borne Rickettsia prowazekii and flea-borne Rickettsia typhi have historically plagued humankind and continue to reemerge globally. The unavailability of vaccines and limited effectiveness of antibiotics late in infection place lethality rates up to 30%, highlighting the need to elucidate the mechanisms of *Rickettsia* pathogenicity in greater detail. Here, we characterize a new effector, Risk1, as a secreted phosphatidylinositol 3-kinase (PI3K) with unique dual class I and class III activities. Risk1 is required for host colonization, and its vacuolar phosphatidylinositol 3-phosphate generation modulates endosomal trafficking to arrest autophagosomal maturation. Collectively, Risk1 facilitates R. typhi growth by altering phosphoinositide metabolism and subverting intracellular trafficking.

## INTRODUCTION

*Rickettsia* species are Gram-negative obligate intracellular bacteria that infect a wide range of eukaryotes, though most notably, blood-feeding arthropods (1, 2). While little is known about numerous ancestral-branching species, three derived *Rickettsia* lineages (spotted fever group [SFG], transitional group [TRG], and typhus group [TG]) are well studied and harbor notable human pathogens (3–5). Tick-borne SFG pathogens, as well as agents of rickettsialpox, Queensland tick typhus, and certain flea-borne diseases (TRG rickettsiae), continue to reemerge in focal areas throughout the world (6). TG pathogens (human body louse-borne Rickettsia prowazekii and flea-borne Rickettsia typhi) have historically plagued humankind and also continue to reemerge globally (7). Infection with R. typhi and *R. prowazekii* results in development of endemic and epidemic typhus, respectively. Both infections present similar clinical manifestations, including high fever, maculopapular rash, headache, and nausea (8). Severe cases develop multiorgan complications such as pneumonia, myocarditis, encephalitis, or meningitis (9). A drastic rise of murine typhus cases alone in Southern California (https://www.cdph.ca.gov/Programs/CID/DCDC/Pages/Typhus.aspx) and Galveston, TX (10), highlights the need for refocusing efforts to combat this serious and underappreciated risk to human health. Importantly, no vaccines are currently available, and misdiagnosis can result in a lethality rate of up to 30% (6).

*Rickettsia* infection into humans occurs either through arthropod blood feeding or inhalation of arthropod feces. Bacteria spread via the lymphatic system and infect a plethora of host cells, including microvascular endothelial cells and immune cells (8). Importantly, as obligate intracellular parasites, *Rickettsia* species replicate in the metabolite-rich host cytosol to complement the depleted rickettsial metabolic circuitry (11). To reach the cytosol of nonprofessional phagocytes, rickettsiae induce host cytoskeletal actin polymerization and plasma membrane (PM) rearrangement (12–14), resulting in a temporary intracellular vacuole that is quickly lysed to avoid lysosomal destruction. Several rickettsial proteins have been implicated in host cell adhesion, invasion, and phagosome escape (15–25). While some of these pathogenicity factors are highly conserved across rickettsial species, others are variably present, indicating species-specific strategies for host cell invasion (1).

Effectors aside, rickettsial secretion systems are highly conserved across species (1). We previously described the *Rickettsia vir* homolog (Rvh) type IV secretion system (T4SS) (26–28) as a translocator of the *Rickettsia* ankyrin repeat protein 2 (RARP-2) and bacterial Sec7-domain containing protein (RalF) effectors (14, 29). These molecules facilitate bacterial infection by modulating endoplasmic reticulum structures and targeting phosphoinositide (PI) metabolism, respectively. As PIs represent a family of signaling lipids that play crucial roles in membrane dynamics and regulating intracellular trafficking (14, 29, 30), we hypothesized that additional rickettsial effectors might target host PI metabolism during invasion. To identify additional T4SS effectors of R. typhi, we used a coimmunoprecipitation approach employing RvhD4 (*Rickettsia* VirD4 homolog), the coupling protein that regulates T4SS effector entry into the secretion channel (14, 26, 29). Bioinformatics and biochemical assays revealed a rickettsial effector phosphatidylinositol 3-kinase (PI3K) with a remarkable dual specificity for phosphoinositides. Functional characterization of this protein, *Rickettsia* intracellular secreted kinase-1 (Risk1), indicates that R. typhi targets host PI pools across multiple membranes throughout infection, including the PM, early endosome, and autophagosome. Collectively, our data suggest that R. typhi establishes host colonization by subverting host intracellular signaling with its minimal effector repertoire.

## RESULTS

### Risk1 is a phosphatidylinositol 3-kinase with class I and III activities critical for Rickettsia typhi invasion.

The characterization of the first R. typhi T4SS effectors, RARP-2 and RalF, indicated that the Rvh T4SS plays a critical role for its intracellular lifestyle (14, 26, 29). We therefore aimed to comprehensively identify new T4SS effectors secreted during R. typhi infection by performing immunoprecipitation (IP) assays using an anti-RvhD4 antibody (αRvhD4 Ab) followed by mass spectrometry analysis (see Fig. S1 in the supplemental material). A bioinformatics pipeline allowed the characterization of seven new putative Rvh T4SS effectors (Fig. S1 and Table S1). The robustness and reliability of our approach was further supported by the identification of other RvhD4 binding partners within the Rvh T4SS machinery (26) and two known secreted effectors, Pat2 (20) and RalF, the latter of which was shown to bind RvhD4 via its C-terminal tail using a bacterial two-hybrid assay (Table S1) (14).

10.1128/mBio.00820-20.1 FIG S1 RvhD4-mediated identification of candidate RvhD4 T4SS effectors. (A) Protein expressed from codon-optimized *RvhD4*_Δ104_ (construct missing the transmembrane segment [TMS] region) was used to generate an αRvhD4 Ab (αRvhD4). The specificity of the Ab was validated by immunoblotting using sucrose-purified R. typhi and lysates of R. typhi-infected and uninfected Vero 76 cells. (B) Immunoprecipitation (IP) was performed on DSP-cross-linked R. typhi-infected lysates using αIgG or αRvhD4 covalently linked magnetic beads. Sample specificity was analyzed by immunoblotting using αRvhD4 Ab. IP reactions were treated with trypsin, analyzed by mass spectrometry. (C) Informatic pipeline for analysis of recovered peptide sequences. The complete list of all analyzed sequences, parsed into spreadsheets, is provided in Table S1. Download FIG S1, EPS file, 1.9 MB. Copyright © 2020 Voss et al. 2020 Voss et al. This content is distributed under the terms of the Creative Commons Attribution 4.0 International license.

10.1128/mBio.00820-20.7 TABLE S1 List of identified candidate RvhD4 T4SS effectors during Rickettsia typhi host invasion. Lysates of R. typhi-infected and uninfected Vero 76 cells were immunoprecipitated with αIgG or αRvhD4 Abs. IP reaction mixtures were treated with trypsin and analyzed by mass spectrometry. Recovered peptide sequences, identified putative effectors, and their predicted domain structures are parsed into spreadsheets. Download Table S1, XLSX file, 0.4 MB. Copyright © 2020 Voss et al. 2020 Voss et al. This content is distributed under the terms of the Creative Commons Attribution 4.0 International license.

**FIG 1:**
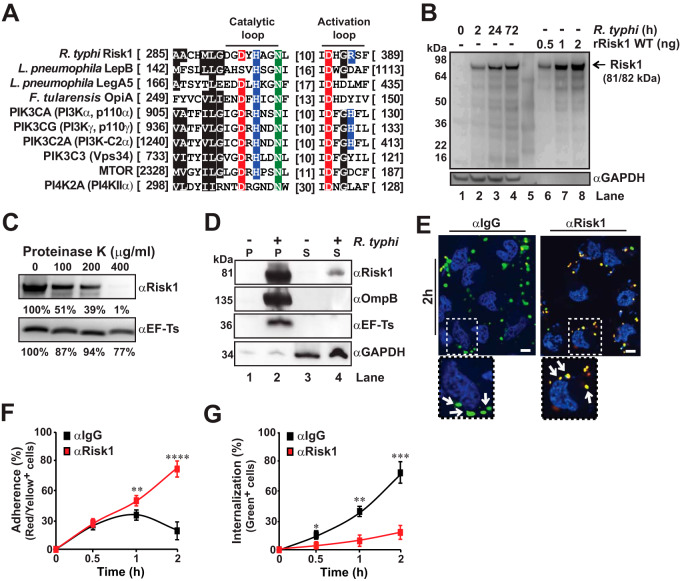
Risk1 is a secreted effector involved in R. typhi host cell invasion. (A) Alignment of Risk1 (RT0135) kinase domain with diverse human and bacterial kinases that carry PI3/PI4-kinase domains. All kinase domains were extracted and aligned using MUSCLE (78) (default parameters). Specific locations of the kinase domains within the selected proteins are displayed in brackets. Amino acids are colored as follows: black, hydrophobic; red, negatively charged; blue, positively charged; green, hydrophilic. (B) Codon-optimized Risk1 protein was used to generate a Risk1 Ab (αRisk1). The specificity of αRisk1 Ab was validated by Western blot analysis using lysates of R. typhi-infected Vero 76 cells (lanes 1 to 4, 0 to 72 h postinfection) and recombinant (r)Risk1 WT protein (lanes 6 to 8, 0.5 to 2 ng). Molecular weights of Risk1 (81 kDa) and rRisk1 (82 kDa) are indicated by the arrow. The minor bands below Risk1 (81/82 kDa) bands may have resulted from degradation of Risk1 or by nonspecific binding. Immunoblotting with αGAPDH Ab was used as equal loading control for lysates of R. typhi-infected Vero 76 cells (lanes 1 to 4). (C) Partially purified R. typhi was treated with increasing concentrations of proteinase K (0 to 400 μg/ml) for 1 h, and lysates were analyzed by immunoblotting for Risk1 and the R. typhi cytoplasmic control EF-Ts. Densitometry was performed using Fiji software, and data are presented as percentage band intensity of proteinase K-treated samples with respect to no treatment. (D) Uninfected or R. typhi-infected Vero 76 cells were lysed with 0.1% Triton X-100 and separated into supernatant (S) and pellet (P) fractions. Samples were analyzed by immunoblotting using αRisk-1, αOmpB, αEF-Ts, and αGAPDH Abs. (E to G) Partially purified R. typhi was preincubated for 0.5 h with αRisk1 (20 μg) or αIgG isotype control Abs (20 μg) and then utilized for HeLa cell infection (MOI, 100:1) for up to 2 h at 34°C. To distinguish between extracellular and engulfed rickettsiae, cells were fixed with 4% PFA and stained first with Alexa Fluor 594-conjugated αR. typhi Ab. Next, cells were permeabilized with saponin and reincubated with Alexa Fluor 488-conjugated αR. typhi Ab. The numbers of adherent (red/yellow) (F) and engulfed (green) (G) bacteria were assessed from 200 cells per well. DNA was stained using DAPI (blue). Bars, 10 μm. Error bars in panels F and G represent means ± SEMs (standard errors of the means) from three independent experiments. **, *P* ≤ 0.01; ***, *P* ≤ 0.005; ****, *P *≤ 0.001.

For one of the identified putative T4SS effectors, NCBI locus tag RT0135, further *in silico* analysis using Phyre2 (31) identified a potential PI3/PI4-kinase domain (pfam00454). Comparison of this domain to those of human PI3Ks (class I, II, or III) and phosphatidylinositol 4-kinase type 2 alpha (PI4K-2α), as well as previously characterized bacterial PI3K (LegA5 and OpiA) and PI4K (LepB) effectors, indicated that RT0135 contains conserved residues within the catalytic and activation loops of this diverse PI3/PI4 family (Fig. 1A). Like most other *Rickettsia* effectors, RT0135 homologs are variably present in different rickettsial species (see Fig. S2A). Despite this, the high similarity across homologs, including a protein from the *Rickettsia* sister lineage (the scrub typhus agent Orientia tsutsugamushi), indicates an important function targeting host cell PIs (Fig. S2B). These collective features prompted the renaming of RT0135 to *Rickettsia* intracellular secreted kinase-1 (Risk1).

10.1128/mBio.00820-20.2 FIG S2 Phylogenomics analysis of Risk1 proteins. (A) Conservation of Risk1 homologs across 89 *Rickettsiaceae* genomes. Risk1 protein sequences were retrieved from blastp searches against the NCBI NR protein database, assembled, and aligned to assess overall conservation (see Materials and Methods). Presence/absence of Risk1 homologs in these 89 genomes was mapped over a previously estimated genome-based phylogeny (79). (B) Alignment of exemplar Risk1 homologs that encompass the full compositional and length diversity. PIK3_4 domain active site features are illustrated as described in the legend for Fig. 1A. GenBank accession numbers as follows: O. tsutsugamushi strain Ikeda (BAG39752), R. bellii OSU 85-389 (ABV79011), R. akari (WP_012013409), R. typhi (WP_011190607), R. prowazekii strain GvV257 (AFE52594), R. montanensis (WP_014409918), R. aeschlimannii (WP_032073887), R. japonica (WP_014120539), R. rickettsii (WP_012150404), and R. conorii (WP_010976857). Download FIG S2, PDF file, 2.0 MB. Copyright © 2020 Voss et al. 2020 Voss et al. This content is distributed under the terms of the Creative Commons Attribution 4.0 International license.

To characterize the functional importance of Risk1 during host infection, we raised an αRisk1 Ab and determined its specificity using R. typhi-infected HeLa cells and recombinant Risk1 protein (Fig. 1B). As the Rvh T4SS lacks T-like pili due to the absence of VirB5-like component on rickettsial surface (1, 28), we tested the hypothesis that translocation of Risk1 through the Rvh-mediated secretion channel deposits the effector on the rickettsial cell surface and, subsequently, delivers Risk1 into the host cytosol during the invasion process. In this effort, we evaluated Risk1 exposure on the R. typhi cell surface by performing a surface digestion assay (Fig. 1C). Indeed, proteinase K treatment of partially purified R. typhi resulted in a dose-dependent degradation of Risk1 on the bacterial membrane compared to that of the rickettsial cytoplasmic control protein, elongation factor Ts (EF-Ts) (Fig. 1C). Next, we sought to demonstrate that Risk1 is secreted into the host cytoplasm during R. typhi infection. In this effort, we performed cellular fractionation of uninfected or R. typhi-infected Vero 76 cells (20) and analyzed the cytoplasmic and pellet fractions by Western blot analysis. We observed that glyceraldehyde-3-phosphate dehydrogenase (GAPDH; host cytoplasmic protein) appeared in the supernatants of both uninfected and infected cells (Fig. 1D, lanes 3 and 4). Of note, the observed faint GAPDH bands within both pellet fractions are likely the result of incomplete lysis of the host cells or residual supernatants left with the pellet fractions (Fig. 1D, lanes 1 and 2). EF-Ts expression was only detectable in the pellet fraction of infected cells (Fig. 1D, lane 2), indicating that our fractionation approach did not result in the lysis of R. typhi. Furthermore, we observed that OmpB, a rickettsial outer membrane protein (32), was only present in the pellet fraction of infected cells (Fig. 1D, lane 2), suggesting that lysis of host cells in the presence of 0.1% Triton X-100 is not affecting the cell surface integrity of R. typhi. Importantly, Risk1 was present in both the pellet (Fig. 1D, lane 2) and supernatant (Fig. 1D, lane 4) of infected cells, implying that Risk1 is secreted into the host cell cytoplasm. Taken together, our data indicate that Risk1 is deposited on the rickettsial cell surface after translocation through the secretion channel and is delivered into the host cell environment during R. typhi infection. Furthermore, Risk1 shows the translocation pattern similar to that of another R. typhi T4SS effector, RalF (14, 28).

To assess the functional importance of Risk1 during R. typhi invasion, we pretreated *Rickettsia* with αRisk1 Ab for various lengths of time and employed differential staining analyses to distinguish between extracellular (tethered) and intracellular bacteria. Our finding showed that neutralization of Risk1 significantly reduced R. typhi internalization, which consequently resulting in an increase in bacterial adherence (Fig. 1E and G). In contrast, pretreatment of R. typhi with an αIgG isotype control Ab showed no reduction in bacterial internalization (Fig. 1E and G), indicating that the reduction in R. typhi infectivity was the result of direct inhibition of Risk1 functionality and not due to steric hindrance induced by the Fc portion of the αRisk1 Ab. These results suggest that Risk1 plays a critical role for R. typhi invasion of host cells.

**FIG 2:**
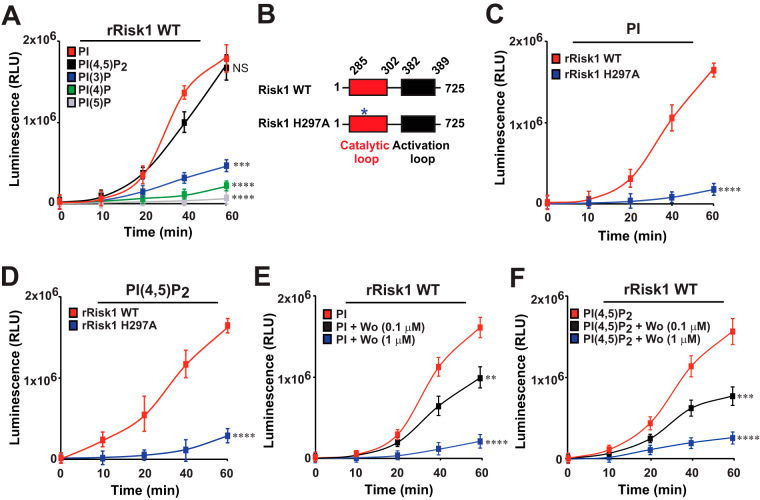
Risk1 functions as a bacterial PI3K with class I and class III activities. (A) Recombinant Risk1 WT protein (rRisk1 WT) was utilized to assess the substrate selectivity by *in vitro* kinase assays using PI, PI(4,5)P_2_, PI(3)P, PI(4)P, or PI(5)P substrates. (B) Site-directed mutagenesis of Risk1 WT was performed to generate the kinase dead mutant, Risk1 H297A. *In vitro* kinase assays of rRisk1 WT and rRisk1 H297A proteins were conducted in the presence of PI (C) or PI(4,5)P_2_ (D). *In vitro* kinase assays were performed using rRisk1 WT plus 1 μM DMSO, rRisk1 WT plus 0.1 μM wortmannin, or rRisk1 WT plus 1 μM wortmannin (Wo) in the presence of both PI (E) or PI(4,5)P_2_ (F). All kinase assay reactions were performed according to the ADP-Glo assay manufacturer’s instructions, and the transfer of phosphates was expressed as relative luminescence units (RLU). Error bars in panels A and C to F represent means ± SEMs from three independent experiments; NS, not significant; **, *P *≤ 0.01; ***, *P *≤ 0.005; ****, *P *≤ 0.001.

The identification of a putative PI3/PI4-kinase domain within Risk1 (Fig. 1A) prompted us to determine whether Risk1 functions as a PI kinase. We therefore examined first the substrate specificity of recombinant full-length wild-type (WT) Risk1 (rRisk1 WT) using a panel of protein-lipid arrays. Risk1 WT bound preferentially to PIs {i.e., phosphatidylinositol [PI], phosphatidylinositol 4-phosphate [PI(4)P], phosphatidylinositol 4,5-bisphosphate [PI(4,5)P_2_], phosphatidylinositol 3,4,5-trisphosphate [PI(3,4,5)P_3_], and phosphatidylserine [PS]} over other lipids such as phosphatidylethanolamine (PE), phosphatidylcholine (PC), diacylglycerol (DAG), cholesterol, or sphingomyelin (see Fig. S3A). The substrate preference for PIs was further validated by performing *in vitro* kinase assays using PI and PI(4,5)P_2_ as the substrates. These molecules were selected based on the substrate specificities of common eukaryotic PI kinases. In general, class I PI3Ks act on PI(4,5)P_2_ to generate PI(3,4,5)P_3_, while class III PI3Ks convert PI to phosphatidylinositol 3-phosphate [PI(3)P] (33). PI4Ks act on PI to generate PI(4)P (34). As anticipated, p110δ/p85α (representing the PI3K group) and PI4K-2α (representing the PI4K group) were able to phosphorylate PI, while only p110δ/p85α additionally phosphorylated PI(4,5)P_2_ (Fig. S3B and C). Strikingly, Risk1 WT was able to phosphorylate both PI and PI(4,5)P_2_ substrates (Fig. 2A and Fig. S3B and C). Furthermore, *in vitro* kinase assays with Risk1 WT using PI(3)P, PI(4)P, and PI(5)P as the substrates revealed extremely low selectivity toward these PIs (Fig. 2A). These data highlight a substrate preferences of Risk1 for PI and PI(4,5)P_2_, a dual PI selectivity previously not observed for other bacterial PI3Ks.

10.1128/mBio.00820-20.3 FIG S3 Risk1 effector phosphorylates both PI and PI(4,5)P_2_ substrates. (A) Lipid membrane assays (Echelon) were performed according to the manufacturer’s instructions. Membrane was spotted with 1 μg of purified rRisk1 WT protein and incubated for 1 h at RT. Binding of Risk1 to phosphoinositides was detected using an αRisk1 and HRP-conjugated Abs. The lipid membrane assay is a representative of three independent experiments. *In vitro* kinase assays were performed using purified rRisk1 WT, human rp110δ/p85α (representing PI3K group), or rPI4K-2α (representing the PI4K group) in the presence of PI (B) or PI(4,5)P_2_ (C). Kinase assays were conducted according to the ADP-Glo assay manufacturer’s instructions, and the transfer of phosphates was expressed as relative luminescence units (RLU). Error bars represent means ± SEMs from three independent experiments. Download FIG S3, EPS file, 0.8 MB. Copyright © 2020 Voss et al. 2020 Voss et al. This content is distributed under the terms of the Creative Commons Attribution 4.0 International license.

The high degree of sequence homology and substrate preference between Risk1 and known PI3Ks (Fig. 1A and 2A and Fig. S3B and C) prompted us to generate a kinase dead mutant (Fig. 2B). A catalytic loop mutant carrying a point mutation at amino acid position 297 (H297A) showed a significant reduction in the kinase activity of Risk1 toward both PI and PI(4,5)P_2_ substrates (Fig. 2C and D). Furthermore, we evaluated the sensitivity of Risk1 to wortmannin, a known PI3K inhibitor (35). As predicted, preincubation with wortmannin significantly inhibited, in a concentration-dependent manner, Risk1’s ability to phosphorylate both PI and PI(4,5)P_2_ substrates (Fig. 2E and F). Collectively, these results suggest Risk1 is a secreted rickettsial PI3K with both class I and class III activities involved in the host cell invasion process of R. typhi.

### The kinase activity of Risk1 modulates cellular phosphoinositide distribution required for Rickettsia typhi host invasion.

**FIG 3:**
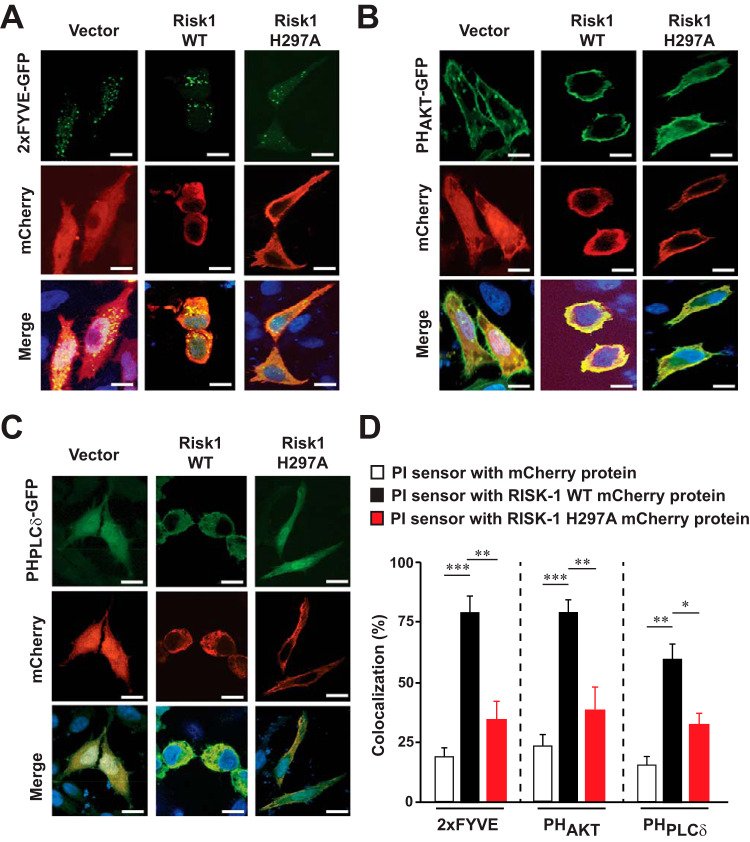
Distribution of phosphoinositides is modulated by Risk1 kinase activity. pmCherry vector, pmCherry-myc-Risk1 WT, or mCherry-myc-Risk1 H297A kinase dead mutant was cotransfected into HeLa cells with fluorescence PI probes for PI(3)P (GFP-2xFYVE) (A), PI(3,4,5)P_3_ and PI(3,4)P_2_ (GFP-PH_AKT_) (B), and PI(4,5)P_2_ (GFP-PH_PLCδ_) (C). Cells were fixed with 4% PFA, and DNA was stained using DAPI (blue). (D) Colocalization patterns of pmCherry alone, pmCherry-Risk1 WT, or pmCherry-myc-Risk1 H297A mutant with the PI probes were analyzed using the Coloc 2 plugin from Fiji software. Graph shows the percentages of pmCherry alone or pmCherry-Risk1 construct colocalization with PI probes. Bars, 10 μm. Error bars represent means ± SEMs from three independent experiments. *, *P *≤ 0.05; **, *P *≤ 0.01; ***, *P *≤ 0.005.

The phosphorylation and dephosphorylation of PIs are key regulatory steps in controlling many cellular processes, including vesicular trafficking. Intracellular bacteria manipulate PI metabolism in order to promote their uptake by target cells, resulting in the establishment of a replicative niche (36). Accordingly, we determined the effects of Risk1 WT and Risk1 H297A on the cellular distribution of various PIs using HeLa cells coexpressing green fluorescent protein (GFP)-tagged PI biosensors (37) with either pmCherry vector, pmCherry-Risk1 WT, or pmCherry-Risk1 H297A. Strong colocalization was observed between Risk1 WT and the biosensors for PI(3)P, PI(3,4,5)P_3_, PI(3,4)P_2_, and PI(4,5)P_2_ (Fig. 3). In contrast, overexpression of Risk1 H297A resulted in a significant reduction in colocalization with all tested PI sensors (Fig. 3), suggesting that Risk1 class I and class III PI3K activities can modulate the distribution of cellular PIs. Based on these findings, we tested whether the kinase activity of Risk1 contributes to host invasion using R. typhi-infected HeLa cells that overexpressed pGFP-vector, pGFP-Risk1 WT, or pGFP-Risk1 H297A. Our findings showed that cells overexpressing Risk1 WT had an ∼2.5-fold higher bacterial burden than cells expressing either vector or Risk1 H297A mutant (see Fig. S4), suggesting that the kinase activity of Risk1 contributes to R. typhi host invasion.

10.1128/mBio.00820-20.4 FIG S4 Risk1 kinase activity is important for R. typhi host invasion. (A and B) HeLa cells were transfected with pGFP-vector, pGFP-Risk1 WT, or pGFP-Risk1 H297A mutant for 12 h followed by incubation with partially purified R. typhi (MOI, 100:1) for an additional 12 h. Cells were fixed with 4% PFA, and R. typhi invasion was detected using αR. typhi and αAlexa Fluor 595 secondary Abs. DNA was stained using DAPI (blue). Bars in panel A, 10 μm. (B) Percentages of bacterial burden were determined from 400 cells using Fiji software. Error bars in panel B represent means ± SEMs from two wells of three independent experiments. **, *P *≤ 0.01. Download FIG S4, EPS file, 2.6 MB. Copyright © 2020 Voss et al. 2020 Voss et al. This content is distributed under the terms of the Creative Commons Attribution 4.0 International license.

### Risk1 targets endosomal trafficking to promote intracellular growth.

**FIG 4:**
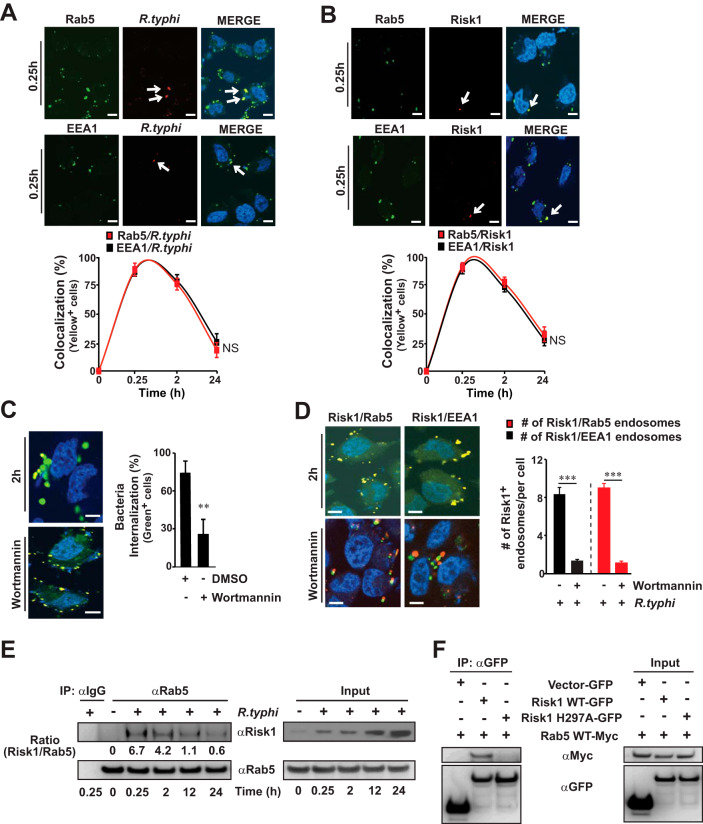
Risk1 associates with endosomal Rab5 and EEA1 during Rickettsia typhi infection. (A and B) HeLa cells were incubated with partially purified R. typhi (MOI, 100:1) for up to 24 h at 34°C. Cells were fixed with 4% PFA, and R. typhi (A) or Risk1 (B) was detected using Alexa Fluor 594-conjugated αR. typhi or Alexa Fluor 594-labeled αRisk1 Abs, respectively. Colocalization with Rab5 or EEA1 was conducted using αRab5 or αEEA1 Abs followed by incubation with an αAlexa Fluor 488 secondary Ab. Graphs show the percentages of R. typhi (A) and Risk1 (B) colocalization with Rab5 and EEA1 (yellow cells) using Coloc 2 plugin Fiji analyzing software. (C and D) HeLa cells were infected with partially purified R. typhi (MOI, 100:1) in the presence of wortmannin (1 μM) or DMSO diluent control for 2 h at 34°C. (C) Cells were stained for engulfed bacteria using Alexa Fluor 594-conjugated αR. typhi Ab followed by permeabilization with saponin and reincubation with Alexa Fluor 488-conjugated αR. typhi Ab. Graph shows the numbers of engulfed (green only) bacteria from 200 cells per well. (D) Expression of Risk1, Rab5, and EEA1 was assessed as described for panel B, and graph shows the numbers of Risk1*^+^* endosomes per cell from at least 200 cells per well. DNA was stained using DAPI (blue). Bars in panels A to D, 10 μm. (E) Lysates of uninfected or R. typhi-infected HeLa cells (MOI, 100:1) were immunoprecipitated (IP) using αRab5 or αIgG-control Abs. Immunoprecipitates and input controls were resolved by SDS-PAGE and immunoblotted with αRab5 or αRisk1 Abs, respectively. Densitometry was performed using Fiji software, and data are presented as fold change between the ratios of Risk1/Rab5. (F) pGFP-vector, pGFP-Risk1 WT, or pGFP-Risk1 H297A mutant was cotransfected into HeLa cells with myc-tagged Rab5 WT. Twelve hours after transfection, cells were immunoprecipitated using an αGFP Ab. Immunoprecipitates and input controls were resolved by SDS-PAGE and immunoblotted with αGFP and αmyc Abs, respectively. Data in panels E and F are a representative of three independent experiments. Error bars shown in panels A to D represent means ± SEMs from two wells of three independent experiments. NS, not significant; **, *P *≤ 0.01; ***, *P* ≤ 0.005.

Species of *Rickettsia* invade host cells by inducing phagocytosis and quickly escaping the phagosomal vacuole to avoid lysosomal degradation, a process previously suggested to involve PI3K-dependent alterations of PI metabolism (38). Given our above-presented data suggesting that Risk1, with its PI3K activities, modulates the distribution of cellular PIs and facilitates R. typhi host invasion, we tested the hypothesis that Risk1 contributes to host invasion by altering endosomal trafficking. In this effort, the cellular localization of Risk1 with two markers of early endosomal vesicle formation (Rab5 and its effector EEA1) were monitored during R. typhi infection. Confocal microscopy analysis showed a time-dependent colocalization pattern between R. typhi and Rab5 or EEA1 (Fig. 4A). Similar results were observed for Risk1 and Rab5 or EEA1 colocalization (Fig. 4B). Next, we treated R. typhi-infected cells with wortmannin, which not only reduced bacterial internalization (Fig. 4C) but also significantly decreased the presence of Risk1 on Rab5- or EEA1-expressing endosomes (Fig. 4D). Furthermore, immunoprecipitation (IP) experiments from R. typhi-infected HeLa cells showed a time-dependent association between secreted Risk1 and endogenously expressed Rab5, reaching a maximum at 0.25 h (∼6-fold greater than at time zero) postinfection (Fig. 4E). To test the hypothesis that complex formation of Risk1 and Rab5 relies on the kinase activity of Risk1, we performed IP experiments from lysates coexpressing myc-tagged Rab5 WT with pGFP-vector, pGFP-Risk1 WT, or pGFP-Risk1 H297A. We observed that binding to Rab5 required the kinase activity of Risk1 (Fig. 4F). These data suggest that inhibition of PI3K activity negatively affects R. typhi internalization and the colocalization of Risk1 with Rab5 and EEA1. Also, our data showing that Rab5 binding to Risk1 requires its kinase activity implies that Risk1 plays a role in the production of PI(3)P for the Rab5-EEA1-PI(3)P-signaling axis.

### Rickettsia typhi subverts autophagy to establish a replicative niche.

Intriguingly, we noticed that transfection with Risk1 WT but not with Risk1 H297A or empty vector resulted in a cellular rounding phenotype (Fig. 3 and Fig. S4). However, we failed to observe membrane blebbing and/or DNA condensation/fragmentation, common hallmarks of cell death (Fig. 3 and Fig. S4) (39). To evaluate this phenotype further, we transfected HeLa cells with pGFP-vector, pGFP-Risk1 WT, or pGFP-Risk1 H297A and quantified the level of cell rounding of only GFP-positive cells. Approximately 75% of Risk1 WT-transfected cells showed cell rounding after 12 h of transfection compared to that of the vector control (see Fig. S5A and B). In contrast, transfection with Risk1 H297A resulted in ∼3-fold lower level of cell rounding, suggesting the phenomenon was dependent on the kinase activity of Risk1 (Fig. S5A and B). Further assessment of apoptosis by flow cytometry using annexin V/7-aminoactinomycin D (7-AAD), and active caspase-3 immunostaining showed no induction of cell death in vector- or Risk1 WT-transfected cells, while cells transfected with Risk1 H297A displayed a significant increase in apoptosis (Fig. S5C). Also, experiments evaluating the proteolytic processing of caspase-3 by Western blot analyses revealed no cleavage of caspase-3 in lysates of Risk1 WT- or vector-transfected cells, while cleavage of caspase-3 was observed in lysates of Risk1 H297A-transfected cells (Fig. S5D). Of note, caspase-3 proteolytic processing in lysates of etoposide-treated HeLa cells (Fig. S5E) were implemented as a positive control. To substantiate our claim that the kinase activity of Risk1 contributes to the phenotype of nonapoptotic cell rounding, we treated Risk1 WT-transfected cells with PI3K inhibitors (LY294002 or wortmannin) and assessed the level of cell death. No induction of apoptosis was observed in dimethyl sulfoxide (DMSO)-treated Risk1 WT-transfected cells or PI3K inhibitor-treated vector-transfected cells (Fig. S5F), while Risk1 WT cells incubated with either wortmannin or LY294002 showed a concentration-dependent increase in the levels of both annexin V/7-AAD and active caspase-3 staining (Fig. S5F). These data indicate that Risk1, via its kinase activity, contributes to a nonapoptotic cell rounding phenotype.

10.1128/mBio.00820-20.5 FIG S5 Risk1 induced nonapoptotic cell rounding relies on its kinase activity. (A and B) HeLa cells were transfected with pGFP-vector, pGFP-Risk1 WT, or pGFP-Risk1 H297A mutant. Cells were fixed with 4% PFA, and changes in cellular morphology (cell rounding) were quantified from 400 counted cells using Fiji software. Boxed regions are enlarged to show detail. Bars, 10 μm. (C) Percentages of annexin V/7-AAD or active-caspase-3 positive staining were assessed 12 h posttransfection on only GFP^+^ HeLa cells described for panel A using flow cytometry. (D) Lysates of HeLa cells described for panel A were immunoblotted with αGFP (used as overexpression control), anti-full-length human caspase-3 (αFL-Caspase-3), anti-cleaved caspase-3 (αCL-Caspase-3), and αGAPDH Abs. (E) HeLa cells were treated with Etoposide (100 μM) or DMSO for an additional 12 h, and lysates were analyzed as described for panel D. (F) HeLa cells were transfected as described for panel A. Six hours after transfections, cells where treated with PI3K inhibitors wortmannin (1 and 5 μM) and LY294002 (1 and 2 μM) or DMSO for an additional 12 h. Percentages of annexin V/7-AAD or active-caspase-3 staining were measured in GFP^+^ cells using flow cytometry. Error bars in panels B, C, and E represent means ± SEMs from three independent experiments. NS, not significant; *, *P* ≤ 0.05; **, *P ≤ *0.01; ***, *P *≤ 0.005. Download FIG S5, EPS file, 1.9 MB. Copyright © 2020 Voss et al. 2020 Voss et al. This content is distributed under the terms of the Creative Commons Attribution 4.0 International license.

**FIG 5:**
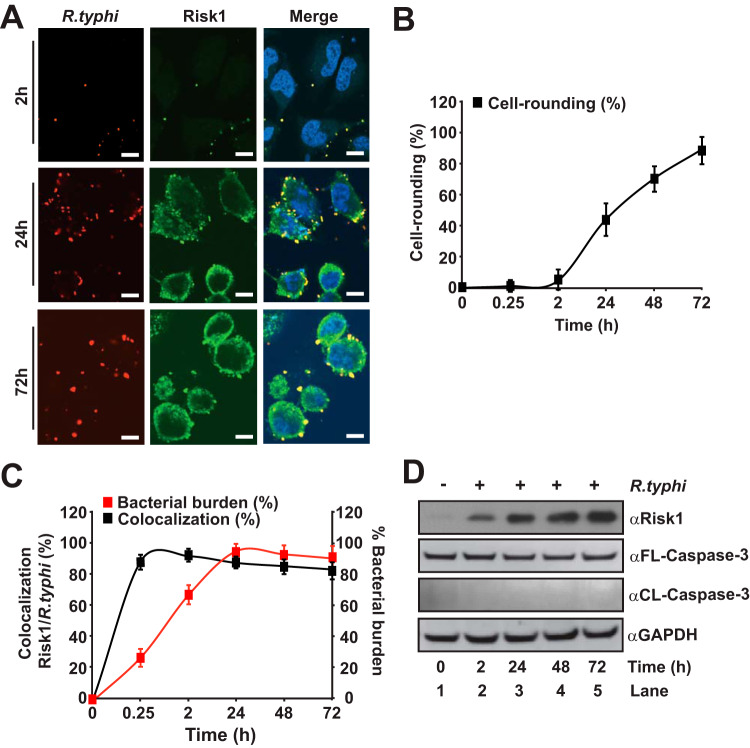
Rickettsia typhi infection induces nonapoptotic cell rounding and increases cytosolic Risk1 expression. (A to D) HeLa cells were incubated with partially purified R. typhi (MOI, 100:1) for various lengths of time (0 to 72 h) at 34°C. Cells were fixed with 4% PFA, and Risk1 expression or R. typhi was detected using αAlexa Fluor 595-conjugated αR. typhi and αAlexa Fluor 488-conjugated αRisk1 Abs. DNA was stained using DAPI (blue). Bars in panel A, 10 μm. (B) Graph shows the number of R. typhi-infected HeLa cells showing rounding was determined from 400 cells. (C) The percentages of cells showing colocalization of R. typhi with Risk1 and the load of bacteria were assessed from experiments performed for panel A. Error bars in panel B and C represent means ± SEMs from two wells of three independent experiments. (D) Lysates of infected HeLa cells described for panel A were immunoblotted with anti-full-length human caspase-3 (αFL-Caspase-3), anti-cleaved caspase-3 (αCL-Caspase-3), αRisk1, and αGAPDH Abs. Immunoblot data are a representative of three independent experiments.

Next, we evaluated whether R. typhi infection results in a similar cell rounding phenotype by conducting confocal microscopy analysis of R. typhi and Risk1 double-stained cells. Similar to that with our Risk1 overexpression approach, R. typhi infection resulted in a time-dependent cellular rounding phenomenon over the course of infection, which correlated with the endogenous expression level of Risk1 and the intracellular growth of the bacteria within the host cytoplasm (Fig. 5A to D). Our data further showed a time-dependent association between R. typhi and Risk1, reaching a maximum after 2 h of infection (Fig. 5C). Notably, R. typhi infection, like Risk1 WT overexpression, did not result in the activation of caspase-3 (Fig. 5D and Fig. S5C and D). Together, our data indicate that cell rounding is a nonapoptotic phenotype induced during R. typhi infection, likely as a result of secreted effector Risk1.

**FIG 6:**
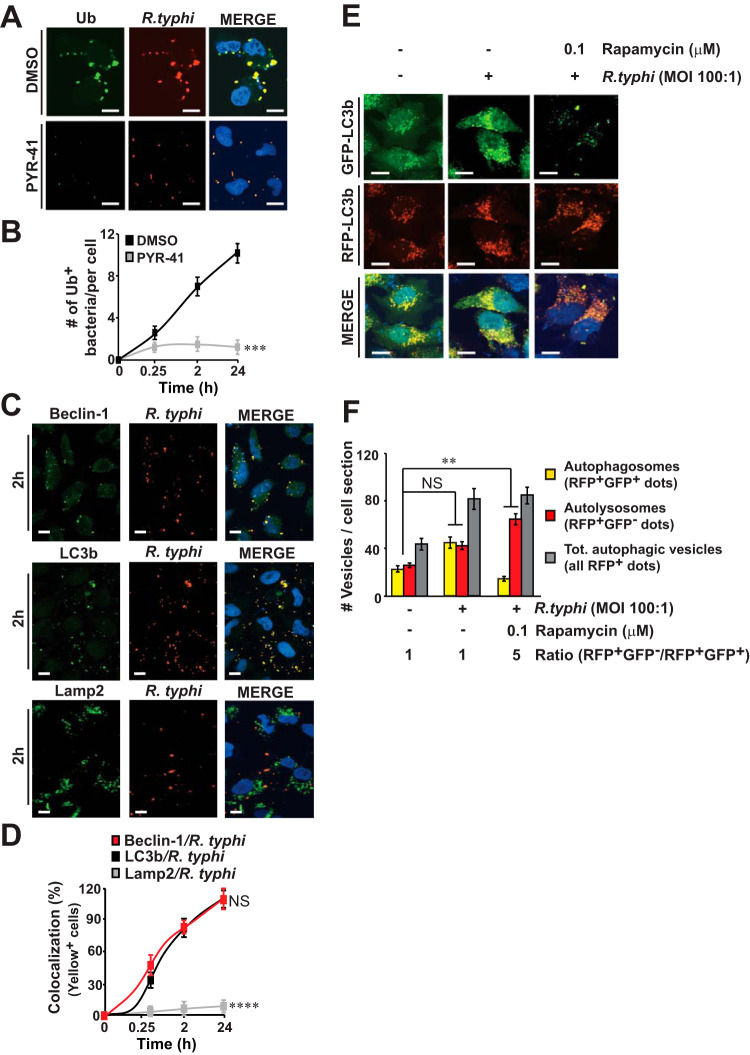
Rickettsia typhi induces autophagy and delays autolysosome formation to establish a replication niche. (A to D) HeLa cells were incubated with partially purified R. typhi (MOI, 100:1) in the presence of ubiquitin-activating enzyme E1 inhibitor PYR-41 (50 μM) or DMSO for various lengths of time at 34°C. (A) Cells were fixed with 4% PFA, and R. typhi was detected using αR. typhi and αAlexa Fluor 594 secondary Abs, while ubiquitination (Ub) was assessed using αUb Ab followed by incubation with an αAlexa Fluor 488 secondary Ab. (B) Graph shows the percentages of cells in which R. typhi colocalized with ubiquitin during the course of infection using Coloc 2 plugin Fiji analyzing software. (C) R. typhi-infected HeLa cells were analyzed for R. typhi as described for panel A, while expression of Beclin-1, LC3b, or LAMP2 was assessed using αBeclin-1, αLC3b, or αLAMP2 Abs followed by an αAlexa Fluor 488 secondary Ab. (D) Graph shows the percentages of cells in which R. typhi colocalized with Beclin-1, LC3b, or LAMP2 using Fiji software. (E and F) R. typhi-infected mRFP-GFP-LC3 HeLa cells were incubated in the presence of rapamycin or DMSO for 2 h at 34°C. R. typhi was detected as described for panel A, and numbers of individual autophagosomes (RFP^+^GFP^+^ dots) and autolysosomes (RFP^+^GFP^−^ dots) per cell were quantified using Fiji software. (F) Results are expressed as absolute numbers of individual vesicles (total autophagic vesicles = all RFP^+^ dots) or presented as fold change ratios of autolysosomes/autophagosomes. DNA was stained using DAPI (blue). Bars in panels A, C, and E, 10 μm. Error bars in panels B, D, and F represent means ± SEMs from three independent experiments. NS, not significant; **, *P *≤ 0.01; ***, *P *≤ 0.005; ****, *P* ≤ 0.001.

Overexpression of PI3K-active Risk1, as well as R. typhi infection, resulting in host cell rounding without inducing apoptosis, a phenotype reminiscent of autophagy induction (40–42). Based on our current data and a preceding report that showed Rickettsia australis induces autophagy to colonize macrophages (43), we tested the hypothesis that R. typhi-induced autophagy avoids autolysosomal destruction to promote bacterial intracellular replication. In this effort, we evaluated if R. typhi is ubiquitylated during host invasion, provided that cytosolic bacteria encounter host ubiquitination prior to recognition by autophagy machinery (44, 45). Our data showed that, unlike Rickettsia parkeri, a member of the SFG (46), R. typhi was ubiquitylated upon host entry, while treatment with ubiquitin-activating enzyme E1 inhibitor, PYR-41, blocked bacterial invasion (Fig. 6A and B and Fig. S6A). Next, we evaluated the status of autophagy adaptor p62/SQSTM1 (47) and autophagic vesicle formation marker LC3 (48) during R. typhi infection. Western blot analyses revealed an increase in autophagic flux as shown by enhanced induction of LC3b (∼3-fold) after 2 h, which remained elevated (∼2.6-fold) after 24 h of infection (Fig. S6B). Furthermore, p62 decreased by 2 h (∼2-fold) and remained downregulated (∼1.8-fold) after 24 h of infection (Fig. S6B). Next, we analyzed upstream signaling events leading to the activation of autophagy and detected an increase in phosphorylation of AMPK at Thr172 (∼2.8-fold) as early as 2 h, which remained elevated (∼3-fold) after 24 h of infection (Fig. S6C). Moreover, phosphorylation of ULK-1 at Ser555, which is critical for the recruitment of Atg13/FIP200 and the initiation of autophagic vesicle formation (49), was induced during the course of R. typhi infection (Fig. S6C), suggesting that R. typhi triggers autophagy through the activation of the AMPK-ULK1 signaling cascade. We expanded on our findings by examining the intracellular localization of R. typhi using autophagosomal makers Beclin-1 and LC3b and the lysosomal marker LAMP2. As predicted, R. typhi colocalizes in a time-dependent manner with Beclin-1 and LC3 but not with LAMP2 (Fig. 6C and D).

10.1128/mBio.00820-20.6 FIG S6 Autophagy induction is critical for Rickettsia typhi host invasion. (A to C) HeLa cells were incubated with partially purified R. typhi (MOI, 100:1) in the presence of E1 inhibitor PYR-41 (50 μM) or DMSO for various lengths of time at 34°C. (A) Cells were fixed with 4% PFA, and R. typhi expression was detected using αR. typhi and αAlexa Fluor 594 secondary Abs, while ubiquitination was assessed using αUb Ab followed by incubation with an αAlexa Fluor 488 secondary Ab. Lysates were immunoblotted with αp62 and αLC3b (B) or αpAMPK-Thr172, αAMPK, αpULK1-Ser555, and αULK1 (C). Immunoblotting with αGAPDH was used as an equal loading control. Western blot analysis is representative of three independent experiments. Densitometry was performed using ImageJ, and data are presented as fold change ratios of p62/GAPDH and LC3b/GAPDH (B) or pAMPK-Thr172/AMPK and pULK1-Ser555/ULK1 (C). R. typhi-infected HeLa cells were incubated in the presence of 3-methyladenine (3-MA; 5 to 10 mM) (D), chloroquine (CQ; 0.1 to 10 μM) (E), or PBS control for 2 h (black bars) or 24 h (red bars) at 34°C. Cells were stained for R. typhi with Alexa Fluor 594-conjugated αR. typhi Ab and then permeabilized with saponin and reincubated with Alexa Fluor 488-conjugated αR. typhi Ab. The numbers of engulfed *Rickettsia* cells (green) per cell were counted for 400 cells per well. DNA was stained using DAPI (blue). Bars in panels A, D, and E, 10 μm. Error bars in panels B to E represent means ± SEMs from three independent experiments. *, *P *≤ 0.05; **, *P *≤ 0.01; ***, *P *≤ 0.005. Download FIG S6, EPS file, 2.5 MB. Copyright © 2020 Voss et al. 2020 Voss et al. This content is distributed under the terms of the Creative Commons Attribution 4.0 International license.

The lack of colocalization between R. typhi and LAMP2 indicated that R. typhi likely avoids fusion with lysosomes during endosomal trafficking. Therefore, we further dissected this mechanism by utilizing two autophagy inhibitors, 3-methyladenine (3-MA) and chloroquine (CQ), for their ability to block autophagy at initiation and autophagosomal maturation stages, respectively (50). Treatment with 3-MA decreased the number of bacteria in a concentration-dependent manner from ∼33% (5 mM) to ∼50% (10 mM) (Fig. S6D) suggesting that initiation of autophagy is required for the intracellular survival of R. typhi. Intriguingly, no reduction in the number of bacteria was observed in the presence of CQ (0.1 to 10 μM) (Fig. S6E, 2 h). However, a prolonged incubation with CQ, but not with 3-MA, showed an increase in R. typhi burden (Fig. S6D and E, 24 h). These findings support the hypothesis that R. typhi induces autophagy during the early stages of invasion, while subsequently avoiding autolysosomal destruction.

To further support our hypothesis that R. typhi subverts autophagosomal maturation, we used mRFP-GFP-LC3-expressing HeLa cells to distinguish between autophagosomes (expressing both red fluorescent protein [RFP] and GFP) and autolysosomes (only express RFP due to the acidified quenching of GFP) (48). Our data revealed that R. typhi infection resulted in an increase in the number of autophagosomes; however, the proportion of autophagosomes and autolysosomes was comparable to the ratio observed in uninfected cells (Fig. 6E and F). Importantly, treatment with rapamycin, an inducer of autophagy flux, resulted in a higher ratio of autolysosomes to autophagosomes within R. typhi-infected HeLa cells than in uninfected or DMSO-treated R. typhi-infected HeLa cells (Fig. 6E and F). Taken together, our data imply that R. typhi-induced autophagy subverts autophagosomal maturation to establish a replicative niche.

### Risk1 contributes to R. typhi-induced autophagy to facilitate intracellular growth.

**FIG 7:**
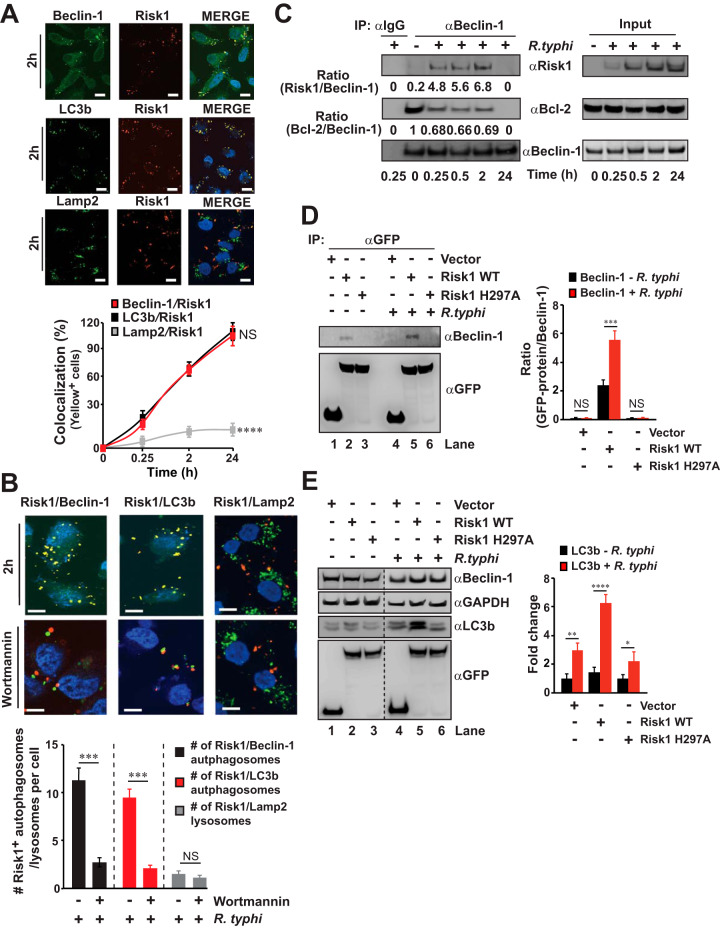
Risk1 is involved in Rickettsia typhi-induced autophagy. HeLa cells were incubated with partially purified R. typhi (MOI, 100:1) in the presence of DMSO (A and B) or wortmannin (B; 1 μM) for 2 h at 34°C. Cells were fixed with 4% PFA, and Risk1 expression was detected using αRisk1 and αAlexa Fluor 594 secondary Abs, while expression of Beclin-1, LC3b, or LAMP2 was assessed using αBeclin-1, αLC3b, or αLAMP2 Abs followed by incubation with an αAlexa Fluor 488 secondary Ab. Graph shown in panel A displays the percentages of cells in which Risk1 colocalized with Beclin-1, LC3b, or LAMP2, while the graph shown in panel B summarizes the numbers of Risk1^+^ endosomes per cell. Data were analyzed using Fiji software from 200 cells per well. DNA was stained using DAPI (blue). Bars in panels A and B, 10 μm. (C) Lysates of uninfected or R. typhi-infected HeLa cells (MOI, 100:1) were immunoprecipitated using αBeclin-1 or αIgG control Abs. Immunoprecipitates and input controls were resolved by SDS-PAGE and immunoblotted with αBeclin-1, αBcl-2, or αRisk1 Abs (D and E) pGFP-vector, pGFP-Risk1 WT, or pGFP-Risk1 H297A mutant was transfected into HeLa cells. Twelve hours after transfection, the cells were incubated with partially purified R. typhi (MOI, 100:1) or left uninfected, and lysates were either immunoprecipitated using an αGFP Ab or resolved by SDS-PAGE followed by immunoblotted with αGFP, αLC3b, αBeclin-1, and GAPDH Abs. Data in panels C to E are a representative of three independent experiments. Densitometry data in panels C to E represent the fold change between the ratios of Risk1/Beclin-1 and Bcl-2/Beclin-1 (C), the binding ratios of Beclin-1 and pGFP-vector or pGFP-Risk1 constructs (D), and the fold change of LC3b (E). Error bars in panels A, B, D, and E represent means ± SEMs from three independent experiments. NS, not significant; *, *P *≤ 0.05; **, *P *≤ 0.01; ***, *P *≤ 0.005; ****, *P *≤ 0.001.

We demonstrated that overexpression of enzymatically active Risk1 resulted in host cell rounding without inducing apoptosis, a phenotype observed during the induction of autophagy (40–42). Intriguingly, our data further revealed that R. typhi induced autophagy and avoided autolysosomal destruction to support intracellular growth. Thus, we tested the hypothesis that Risk1 plays a role in R. typhi-induced autophagy. In this effort, we examined the subcellular localization of Risk1 during R. typhi infection. Similar to R. typhi data (Fig. 6C and D), we observed a time-dependent colocalization between Risk1 and Beclin-1 or LC3b, but not with LAMP2 (Fig. 7A). Next, we observed that the treatment with the PI3K inhibitor, wortmannin, blocked that colocalization of Risk1 with both autophagy markers (Beclin-1 or LC3b) (Fig. 7B). Of note, wortmannin treatment did not alter the localization of Risk1 and LAMP2 (Fig. 7B). These data imply a role of the PI3K activity in the colocalization of Risk1 with both autophagy markers (Beclin-1 and LC3b).

Autophagy induction involves activation of the Beclin-1-Vsp34-Vsp15 core complex via the dissociation of its negative regulator Bcl-2 from Beclin-1 (45, 51–54). Accordingly, we tested the hypothesis that Risk1, which exhibited class III PI3K activity, contributes to autophagy induction by modulating the Beclin-1-Bcl-2 complex. Using IP assays, we demonstrated a time-dependent association between R. typhi effector Risk1 and host Beclin-1, reaching a maximum at 2 h (∼6-fold greater than at time zero) postinfection (Fig. 7C). Moreover, our data revealed a time-dependent dissociation of Bcl-2 from Beclin-1 during R. typhi infection (Fig. 7C). No association between Beclin-1 and Risk1 or Bcl-2 was observed after prolonged R. typhi infection (Fig. 7C, 24 h).

Our data revealed that Beclin-1 binds to Risk1; thus, we tested the hypothesis that Beclin-1 binding requires the kinase activity of Risk1. In this effort, we performed IP experiments from lysates of HeLa cells expressing pGFP-vector, pGFP-Risk1 WT, or pGFP-Risk1 H297A with or without R. typhi infection. Our data show that Beclin-1 only associated with Risk1 WT in uninfected cell lysates, which was significantly enhanced upon R. typhi infection (Fig. 7D), suggesting that Beclin-1 binds only the enzymatically active Risk1 effector. Next, we tested the hypothesis that the PI3K activity of Risk1 contributes to R. typhi-induced autophagy by evaluating the autophagic flux as measured by LC3b induction in HeLa cells expressing pGFP-vector, pGFP-Risk1 WT, or pGFP-Risk1 H297A with or without R. typhi infection. Western blot analyses showed no changes in the LC3b induction for uninfected HeLa cells expressing pGFP-vector, pGFP-Risk1 WT, or pGFP-Risk1 H297A (Fig. 7E). However, with R. typhi infection, we observed an ∼2- to 3-fold increase in LC3b induction in HeLa cells expressing pGFP-vector or pGFP-Risk1 H297 compared to that in samples of uninfected cells (Fig. 7E), which was further elevated to ∼6-fold in R. typhi-infected HeLa cells expressing pGFP-Risk1 WT (Fig. 7E). Taken together, our data suggest PI3K active Risk1 binds with Beclin-1 of the class III PI3K core complex (Beclin-1-Vsp34-Vsp15) and plays a role in autophagy induction, as measured by the LC3b marker, during R. typhi infection.

Collectively, these data indicate that Risk1 is a multifunctional PI3K, which modulates intracellular trafficking to facilitate the cytosolic survival of R. typhi.

## DISCUSSION

Intracellular bacteria have developed numerous strategies to avoid host microbicidal defense mechanisms to establish a replicative niche (8, 52, 55, 56). One such approach entails reprogramming host PI metabolism, which can facilitate uptake into host cells, modify phagosomes, undermine apoptosis, and interfere with other cellular defense mechanisms (57). Specifically, certain bacteria possess eukaryotic-like PI kinases and phosphatases that modulate PI concentrations at specific membrane foci, altering temporal and spatial regulation of host signaling transduction and protein recruitment to membranes (58). To date, PI kinases have been characterized in two well-studied intracellular pathogens. Legionella pneumophila evades endosomal degradation through the secretion of the PI4K LepB and the 3-phosphatase SidF, two *dot*/*icm* T4SS effectors that contribute to the synthesis of PI(4)P on the *Legionella*-containing vacuole (59). Alternatively, Francisella tularensis secretes the T6SS effector protein OpiA, a PI3K that promotes the production of PI(3)P on *Francisella-*containing phagosomes to prevent endosomal maturation and facilitates the escape from the phagosome (60). Another *dot*/*icm* effector from L. pneumophila, named LegA5, was characterized alongside OpiA as a similar class III PI3K, consistent with numerous PI-interacting *dot*/*icm* effectors functioning to support *Legionella* vacuolar growth and survival (61). Like L. pneumophila and F. tularensis, survival of *Rickettsia* species involves the avoidance of endolysosomal fusion (55). While the precise mechanisms by which *Rickettsia* species avoid lysosomal degradation remain largely unknown, we reasoned that similar PI-mediated mechanisms employed by bacteria such as L. pneumophila and F. tularensis were likely encoded in rickettsial genomes.

In our prior reports, we showed the R. typhi effector RalF activates Arf6, which in turn recruits host phosphatidylinositol 4-phosphate 5-kinase (PIP5K) for conversion of PI(4)P to PI(4,5)P_2_, suggesting that R. typhi effectors initiate alterations in PI metabolism to facilitate host cell invasion (14, 38). As RalF was shown to be a T4SS effector, we posited that other Rvh effectors might also function in modulating host cell PI metabolism. In this study, we evaluated the RvhD4 interactome and identified a rickettsial conserved hypothetical protein (RT0135) that we named Risk1 along with seven putative RvhD4 effectors and several known VirD4-binding partners (see Table S1 in the supplemental material). Our informatics analysis revealed that Risk1 contains a kinase active site conserved across human PI3Ks (class I, II, or III), human PI4K-2α, and three bacterial effectors (LepB, LegA5, and OpiA). Further biochemical and enzymatic assays indicated Risk1 is a PI3K with specificity for both PI and PI(4,5)P_2_, making it the first bacterial PI3K with class I and class III activities.

**FIG 8:**
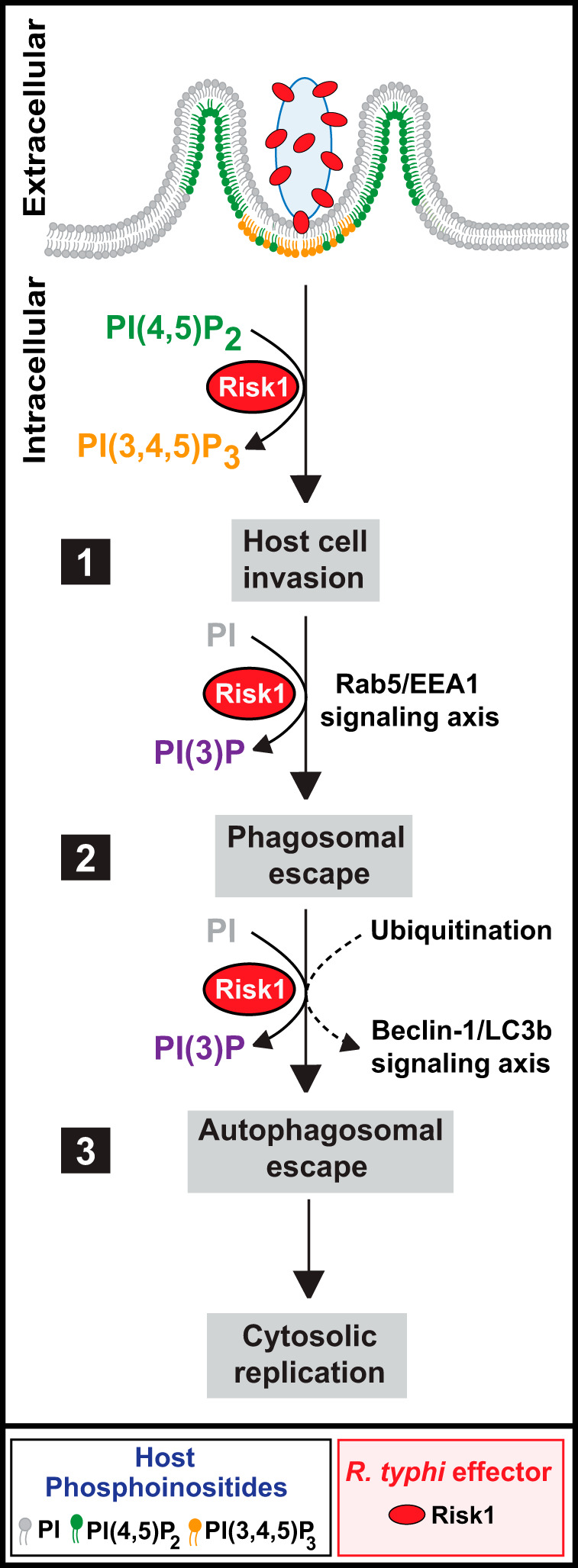
Working model for Risk1 promoting Rickettsia typhi intracellular survival. The proposed model for Risk1 consists of three main conceptual stages: host cell invasion (1), phagosomal escape (2), and autophagosomal escape (3). (Step 1) Secreted Risk1 facilitates host cell invasion by converting PI(4,5)P_2_ to PI(3,4,5)P_3_. (Step 2) Risk1 likely plays a role in early endosome formation by targeting the Rab5-EEA1-PI(3)P signaling axis. (Step 3) After phagosomal escape, R. typhi becomes ubiquitinated, resulting in the initiation of autophagy, which is likely facilitated by the binding of Risk1 to host Beclin-1. Finally, we propose that the observed delay in fusion of phagosomes/autophagosomes with lysosomes is likely due to the accumulation of PI(3)P by Risk1, leading to the observed escape from phagosomes/autophagosomes to establish a replicative niche.

Previous work suggested that PI3K-mediated PI metabolism plays a role in *Rickettsia* infection. In particular, pharmacological inhibition of PI3Ks revealed PI(3,4,5)P_3_ synthesis was important for R. conorii infection, another member of the SFG (12), while our work showed PI3K-dependent synthesis of both PI(3)P and PI(3,4,5)P_3_ is critical during R. typhi phagocytosis and endosomal escape (38). However, the source of this PI3K (i.e., host and/or rickettsial) and the precise mechanism of its action remained unknown. Using confocal microscopy and biochemical assays, we found that colocalization of Risk1 with both early endosomal markers Rab5 and EEA1 required the PI3K activity of Risk1. Importantly, experiments using cells expressing biosensors for PI(4,5)P_2_, PI(3,4,5)P_3_, or PI(3)P identified Risk1 as a PI3K capable of targeting various cellular PI pools. Collectively, these data position Risk1, and particularly its dual class I and class III properties, as the likely source for PI3K activity that subverts host PI metabolism to facilitate R. typhi internalization and escape into the host cytosol before endolysosomal destruction (Fig. 8, steps 1 and 2, respectively). The latter role in cytosolic access is predicted to coincide with the action of membranolytic Pat1 and Pat2 phospholipase effectors, which we previously reported to be important for R. typhi intracellular survival (19, 20).

Aside from endolysosomal destruction, the survival of intracellular pathogens is also challenged by autophagy. A process for orderly degradation and recycling of cellular components, autophagy becomes an arm of the cellular innate immune system (also termed xenophagy), directed against invading pathogens (52). Many pathogens have evolved strategies to block autophagy and/or subvert the machinery to support their intracellular survival (44, 62). For instance, Shigella flexneri induces autophagy upon entry into the host cell through the binding of its own surface protein VirG to Atg5 (63). However, *Shigella* avoids autophagolysosomal degradation through its T3SS effector IcsB, which competitively binds to VirG resulting in the disruption of the Atg5/VirG complex (63). Additionally, *Shigella* proteins IcsB and VirA have been shown to facilitate the escape of S. flexneri from Atg8/LC3-positive vacuoles during cell-to-cell spread of the bacteria (64). Another intracellular pathogen, Listeria monocytogenes, escapes autophagic recognition through the interaction of the ActA effector with the cytosolic actin polymerization machinery (ARP2/3, VASP, and actin), thereby inhibiting the bacterial association with ubiquitin and p62 (65). More recent findings showed that *Listeria* utilized ActA in conjunction with two phospholipases (PlcA and PlcB) to avoid autophagy (66).

In the case of *Rickettsia* species, recent reports revealed conflicting results on autophagy induction by SFG rickettsiae during host invasion (43, 46). *R. australis*, a member of SFG rickettsiae, was reported to benefit from autophagy induction for bacterial growth (43), while *R. parkeri*, a member of the same SFG rickettsiae, blocks ubiquitylation and subsequently avoids autophagy induction for its survival (46). Our current data on a member of TG rickettsiae showed that R. typhi was ubiquitylated upon host entry and that ubiquitylation was critical for R. typhi invasion. Moreover, we demonstrated that R. typhi promoted autophagy during host invasion and further demonstrated that autophagy was important for intracellular survival. Thus, our findings are in line with recent reports, which show that *R. australis* induced autophagy for successful host invasion (43). In this study, we demonstrated that R. typhi and its secreted effector Risk1 colocalize with LC3b and Beclin-1. Furthermore, our data revealed that Risk1, via its PI3K activity, facilitates binding to Beclin-1 and enhances R. typhi infection, suggesting that R. typhi utilizes early autophagosome formation as part of its successful host invasion strategy (Fig. 8, step 3). This mechanism seems fairly consistent with rickettsial relatives Anaplasma phagocytophilum and Ehrlichia chaffeensis, which utilize different effectors to target Beclin-1 and reroute autophagosome cargo to vacuoles harboring these bacteria (67). However, this “nutritional virulence” model is harder to envision for *Rickettsia* species, which do not replicate within modified phagosomes. For Orientia tsutsugamushi, another rickettsial species that lyses the phagosome and replicates freely in the host cytosol, autophagic recognition is outright avoided despite autophagy induction during infection (68). These data for diverse rickettsial species accentuate the divergent strategies utilized across *Rickettsiales* for intracellular parasitism (69).

Nutritional virulence aside, our data suggest that R. typhi subverts autophagosome maturation likely in a Risk1-mediated process. Pharmacological targeting of autophagy using 3-MA or wortmannin, two PI3K inhibitors that target the PI3K class III complex controlling the initiation/elongation phase, inhibited R. typhi infection as well as autophagic flux. In contrast, chloroquine, a common antimalaria drug that inhibits autophagosomal fusion with lysosomes, failed to block R. typhi infection. In fact, prolonged chloroquine treatment (24 h postinfection) increased R. typhi burden, suggesting that a delay in autophagosomal maturation through a PI3K-dependent mechanism is facilitating R. typhi escape from autolysosomal destruction for survival. Further interrogation of this mechanism using mRFP-GFP-LC3-expressing HeLa cells showed that infection with R. typhi resulted in a comparable autophagosome-to-autolysosome ratio, implying that R. typhi abates autophagic maturation. Such a process is shared with several other bacterial species, including Mycobacterium marinum (70), Chlamydia trachomatis (71, 72), Yersinia pestis (73), and Francisella tularensis (74). Therefore, it is tempting to speculate that the autophagic uptake of R. typhi represents a purposely induced mechanism to provide a survival advantage to the bacteria. One likely possibility is that autophagy induction allows R. typhi to elute inflammasome-dependent recognition, and thereby its distraction, through the anti-inflammatory effects elicited by the autophagy machinery (44, 45, 62, 75); however, the precise mechanism remains to be defined.

In this study, we provided supporting evidence that Risk1 possibly functions as a modulator of R. typhi-induced autophagy through its association with Beclin-1. In addition, we characterized Risk1 as a secreted effector with class III PI3K activity converting PI to PI(3)P to likely delay lysosomal fusion. Given that the consumption of PI(3)P on either endosomal or autophagosomal membranes is a prerequisite for their fusion with lysosomes, it is tempting to propose a conceptual model of rickettsial cytosolic infection by which Risk1 is the enzyme responsible for the generation of PI(3)P on both the phagosomal and autophagosomal membranes to delay their maturation (60). In turn, the delay in maturation of these structures would allow other effectors, such as Pat1 and Pat2 phospholipases, to perforate their membranes to mediate bacterial escape into the cytosol (Fig. 8, steps 2 and 3).

In sum, our data suggest that the R. typhi-secreted effector Risk1, with class I and class III PI3K activities, facilitates intracellular growth by altering PI metabolism during internalization and subsequently subverts autophagosomal maturation to promote intracellular growth (Fig. 8). In contrast to other intracellular pathogens with enormous effector arsenals (i.e., L. pneumophila and its bevy of *dot*/*icm* effectors), this versatile effector provides insight on how an obligate intracellular pathogen with a highly reductive genome (and hence minimal effector repertoire) effectively overrides the host intracellular signaling program to efficiently colonize the intracellular environment.

## MATERIALS AND METHODS

### Antibodies and reagents.

Beclin-1 (mouse, 2A4; rabbit, D40C5), GFP (rabbit, D5.1), LC3b (E5Q2K), Rab5 (E6N8S), and cleaved caspase-3 Abs were from Cell Signaling Technology. EEA-1 and full-length caspase-3 Abs were from BD Transduction Laboratory. LAMP2 (H4B4), GAPDH (FL-335), GFP (mouse, B-2), c-Myc (9E10), Bcl-2 (C-2), and horseradish peroxidase (HRP)-conjugated secondary Abs (αmouse, αrabbit, αrat, and αgoat IgGs) were from Santa Cruz Biotechnology. Mono- and polyubiquitinylated conjugated antibodies (FK2) (αUb) were obtained from Enzo Life Sciences. The p62/SQSTM1 Ab, chloroquine, 3-methyladenine, rapamycin, PYR-41 (ubiquitin-activating enzyme E1 inhibitor), proteinase K, and lysozyme (from hen egg white) were purchased from Sigma. ProLong Gold antifade mounting medium with DAPI (4′,6-diamidino-2-phenylindole), paraformaldehyde (PFA), Halt protease and phosphatase inhibitor cocktail, Dynabeads (M-270 Epoxy and A/G-agarose), dithiobis(succinimidyl propionate) (DSP), and Alexa 488/594-conjugated secondary Abs were purchased from Thermo Fisher Scientific. Recombinant phosphatidylinositol 3-kinase p110δ/p85α, phosphatidylinositol 4-kinase type 2 alpha (PI4K-2α), phosphatidylserine, and all utilized phosphoinositides were purchased from Echelon Biosciences. The PI3K inhibitors, wortmannin and LY294002 were obtained from Calbiochem and dissolved in the diluent dimethyl sulfoxide (DMSO; Sigma).

### Bacterial strains, cell culture, and infection.

Vero 76 (African green monkey kidney, RL-1587; ATCC) and HeLa (CCL-2; ATCC) cells were maintained in minimal Dulbecco’s modified Eagle’s medium (DMEM) supplemented with 10% heat-inactivated fetal bovine serum (FBS) at 37°C with 5% CO_2_. R. typhi strain Wilmington (obtained from the CDC) was propagated in Vero 76 cells grown in DMEM supplemented with 5% FBS at 34°C with 5% CO_2_. R. typhi was purified as previously described (76). For host cell infections, R. typhi was used at a multiplicity of infection (MOI) of 100:1. For PI3K inhibitor assays, cells were washed with DMEM with 5% FBS prior to infection and pretreated for 2 h with wortmannin, LY294002, or equal volumes of DMSO.

### Identifying putative RvhD4 T4SS effectors.

For capturing RvhD4 effectors, a codon-optimized RvhD4 gene lacking the region encoding the N-terminal VirD4-like transmembrane-spanning domain (*rvhD4*_Δ104_) was used to generate an anti-RvhD4 Ab (αRvhD4 [29]). The specificity was validated by immunoblotting using lysates of R. typhi-infected Vero 76 cells (see Fig. S1A in the supplemental material). For IP experiments, we first collected cellular material from five T150 flasks of R. typhi*-*infected Vero 76 cells (96 h postinfection), which then were partially purified by double sucrose cushion and resuspended in 1× phosphate-buffered saline (PBS). Samples were cross-linked with 80 μl of 25 mM DSP, incubated on ice for 2 h, and then mixed with 20 μl Tris-HCl (pH 7.5) for 15 min at room temperature (RT) to stop the reaction. Cross-linked rickettsiae were washed extensively in 1× PBS and resuspended in IP buffer (Dynabeads coimmunoprecipitation kit; Thermo Fisher Scientific) containing protease inhibitor mini EDTA-free, 100 mM NaCl, and 1 mg/ml lysozyme. Reaction mixtures were incubated on ice for 30 min, sonicated (6.5 setting) for 3 × 30 s on ice, and then centrifuged at 16,000 × *g* for 10 min at 4°C. Cross-linked rickettsial supernatants were collected and stored at −80°C for the IP assay. Next, an excess of αIgG or αRvhD4 Ab (25 μg per mg of beads) was used to cross-link 7.5 mg of 2.8-μm Dynabeads M-270 epoxy beads. Beads were washed and resuspended in extraction buffer according to the manufacturer’s instructions. The αIgG or αRvhD4 Ab-coated beads were mixed with the cross-linked rickettsial supernatants, and reaction mixtures were incubated for 30 min at 4°C. Immunoprecipitated samples were sent for trypsin digestion and mass spectrometry analysis at the UMB protein analysis core facility. Recovered peptides were culled of singletons and Vero 76 cell proteins, with further ranking by sequence coverage and informatics scrutinization resulting in seven new and two already known candidate Rvh effectors (Table S1).

### Bioinformatics and phylogenomics analyses.

Preliminary sequence analyses of Risk1 using blastp (against the NCBI Conserved Domains Database) (77) and Phyre2 (31) indicate Risk1 contains the catalytic and activation loops that define characterized bacterial and human PI3, PI4, and certain protein kinases (pfam00454, PI3_PI4_kinase). The putative Risk1 active site of R. typhi strain Wilmington (AAU03620) excised from the full-length protein sequence and was aligned to analogous regions within bacterial PI4 (LepB of L. pneumophila subsp. *pneumophila* strain Philadelphia 1, WP_010948192), PI3 (LegA5 of L. pneumophila, WP_010948028, and OpiA of F. tularensis subsp. *novicida* strain U112, ABK89042), as well as human class I (PIK3CA, NP_006209.2, and PIK3CG, NP_002640.2), class II (PIK3C2A, NP_002636.2), class III (PIK3C3, NP_002638.2), and class IV (mTOR, NP_004949) PI3Ks, and a selected human PI4K (PI4K2A, NP_060895). Protein domains were aligned using MUSCLE (default parameters) (31). Phylogenomics analysis of Risk1 homologs across 89 *Rickettsiaceae* genomes (87 *Rickettsia* genomes and two O. tsutsugamushi genomes) initiated by retrieving proteins from NCBI using R. typhi strain Wilmington Risk1 (locus tag RT0135) as the query in blastp searches against the NR (All GenBank plus RefSeq nucleotides plus EMBL plus DDBJ plus PDB) database, coupled with a search against the Conserved Domains Database (77). Searches were performed with composition-based statistics, with no filter used. Default matrix parameters (BLOSUM62) and gap costs (existence, 11; extension, 1) were implemented, with an inclusion threshold of 0.005. Subjects were aligned using MUSCLE with default parameters (78). Presence/absence of Risk1 homologs in these 89 genomes were mapped over a previously estimated genome-based phylogeny (Fig. S2A) (79). A smaller alignment containing exemplar genomes that encompass the full sequence compositional and length diversity is shown in Fig. S2B.

### Recombinant proteins and antibody against rickettsial antigen.

Codon-optimized recombinant proteins for wild-type (WT) full-length Risk1 (Risk1 WT), and the catalytically dead mutant (Risk1 H297A) were expressed and purified by GenScript (Piscataway, NJ). Rabbit Abs against recombinant Risk1 (αRisk1) or OmpB (αOmpB) proteins were generated and affinity purified by GenScript. The specificity of αRisk1 or αOmpB was validated by immunoblotting. Antibodies against cytoplasmic housekeeping protein elongation factor Ts (EF-Ts) were obtained from Primm Biotech, Inc., Cambridge, MA, as described previously (19).

### Mammalian expression plasmids.

Plasmids encoding green fluorescent protein (GFP) or mCherry-tagged codon-optimized full-length Risk1 WT and Risk1 H297A mutant were generated by GenScript. pGFP-2xFYVE was a kind gift from George Banting (80). The pGFP-PH_AKT_ plasmid was a kind gift from Craig Montell (Addgene plasmid 18836) (81). The pGFP-PH_PLCδ_ construct (Addgene plasmid 21179) was kindly gifted by Tobias Meyer (82).

### Extract preparation, immunoprecipitation, and Western blot analysis.

*Rickettsia*-infected HeLa cells were lysed for 2 h at 4°C in ice-cold lysis buffer (50 mM HEPES [pH 7.4], 137 mM NaCl, 10% glycerol, 1 mM EDTA, 0.5% NP-40, and supplemented with protease and phosphatase inhibitory cocktails). Cellular debris were removed by centrifugation at 16,000 × *g* for 15 min at 4°C. Equal amounts of protein, as determined by Bradford assay, were loaded for SDS-PAGE and then transferred onto polyvinylidene difluoride (PVDF) membranes. Membranes were probed with Abs of interest, followed by enhanced chemiluminescence with secondary Abs conjugated to horseradish peroxidase. For immunoprecipitation of endogenous proteins, 2 mg of each lysate was immunoprecipitated overnight at 4°C with Abs αRab5, αBeclin-1, or αIgG isotype as a control, followed by 2 h of incubation with 15 μl of protein G-agarose Dynabeads. Immunoprecipitates were washed three times with lysis buffer, and the reactions were analyzed by immunoblotting. For experiments using lysates of cells overexpressing pGFP-vector, pGFP-Risk1 WT, or pGFP-Risk1 H297A mutant in the presence or absence of myc-tagged Rab5 WT, 0.5 mg of each lysate was immunoprecipitated overnight at 4°C with an αGFP Ab and incubated for additional 2 h with G-agarose Dynabeads. Samples were processed and analyzed as described above.

### Protease treatment of R. typhi.

Assessment of surface exposure of Risk1 was performed as described previously (38). Briefly, R. typhi-infected Vero 76 cells were resuspended in 1× PBS containing MgCl_2_ (1× PBS-Mg) and sonicated for 10 s on ice to release R. typhi from host cells. The suspension was filtered through a 5-μm filter, and the filtrate was layered onto a 20% sucrose cushion at a 1:1 ratio and centrifuged at 16,000 × *g* for 15 min at 4°C. The R. typhi-containing pellet was resuspended in 1× PBS-Mg and further purified with a 20% sucrose cushion. Purified R. typhi was treated with various concentration of proteinase K for 1 h at RT in 1× PBS-Mg buffer, and the proteinase reaction was stopped by the addition of Halt protease and phosphatase inhibitor cocktail. Bacteria were again centrifuged at 16,000 × *g* for 10 min at 4°C, resuspended in 1× PBS-Mg, and Risk1 as well as EF-Ts expression was analyzed by immunoblotting. Densitometry was performed using Fiji (ImageJ) software (NIH).

### Secretion assay.

Vero 76 cells, either infected or uninfected with R. typhi, were incubated in culture medium at 34°C as described elsewhere (20). Briefly, cells were lysed in 1× PBS buffer (containing 0.1% Triton X-100, protease and phosphatase inhibitors) for 0.5 h on ice (83). Lysates were centrifuged for 10 min at 10,000 × *g* to separate the rickettsial secreted effectors and host cytosolic proteins (supernatant fraction) from the intact rickettsiae and insoluble host proteins (pellet fraction). The supernatant fraction was filtered through a 0.45-μm-pore-size filter (Millipore), and proteins were precipitated using one-tenth volume of trichloroacetic acid and 1/100 volume of 2% sodium deoxycholate overnight at 4°C. The precipitated proteins of the supernatant fraction were pelleted by centrifugation at 16,000 × *g* for 10 min at 4°C, followed by washing with cold acetone. Samples from the pellet and supernatant were immunoblotted with αRisk1, αOmpB (as control for R. typhi surface protein), αEF-Ts (as control for R. typhi cytoplasmic protein), or αGAPDH Abs (host cytoplasmic control protein) (20).

### *In vitro* kinase and lipid membrane assays.

*In vitro* kinase assays were performed using the ADP-Glo kinase assay kit according to the manufacturer’s instructions (Promega). Briefly, phosphoinositides were dissolved in water at a concentration of 1 mM in the presence of phosphatidylserine at a ratio of 1:3. The kinase reaction was mixed with the kinase buffer (50 mM HEPES [pH 7.5], 50 mM NaCl, 5 mM MgCl_2_, 1 mM dithiothreitol [DTT], and 0.025 ng/ml bovine serum albumin [BSA]) and incubated at RT for up to 1 h in the dark. Reactions were stopped by adding 25 μl of ADP-Glo reagent. After 40 min of incubation at RT, 50 μl of the kinase detection reagent was added, and the plates were incubated for additional 1 h at RT. For wortmannin inhibition assays, purified rRisk1 WT protein was incubated with different concentrations of wortmannin or equivalent volumes of DMSO, and the kinase reaction was then performed as described above. The transfer of phosphates was expressed as relative luminescence units (RLU). Luminescence was measured using the Omega Multimode plate reader.

Lipid membrane assays were obtained from Echelon (P-6002, P-6003) and performed according to the manufacturer’s instructions. Briefly, 1 μg of purified rRisk1 WT protein was spotted onto the membrane and incubated for 1 h at RT. Membranes where washed using 1× PBS-Tween 20 (0.1%), and membranes were probed with an αRisk1 Ab for 1 h at RT followed by another incubation period of 1 h with HRP-conjugated antibodies. Membranes where washed and exposed. The lipid membrane assay was repeated three times.

### Immunofluorescence.

Eight-well chamber slides were seeded with HeLa cells and infected with partially purified R. typhi as described previously (14). Brief, partially purified rickettsiae were added to HeLa cells and incubated for various length of time at 34°C with 5% CO_2_. Following incubation, cells were washed three times with 1× PBS and fixed with 4% paraformaldehyde (PFA) for 20 min at RT. Cells were than permeabilized in blocking buffer (0.3% saponin and 0.5% normal goat serum in 1× PBS) for 30 min and incubated for 1 h with the following primary Abs diluted in Ab dilution buffer (0.3% saponin in 1× PBS): αR. typhi (1:500), αRisk1 (1:500), αUb (1:100), αBeclin-1 (1:100), αLC3b (1:100), αLAMP2 (1:100), αRab5 (1:100), and αEEA1 (1:100). Cells were then washed with 1× PBS and incubated for 1 h with αAlexa Fluor 594 or αAlexa Fluor 488 secondary Abs diluted 1:1,500 in Ab dilution buffer. For Risk1 kinase neutralization experiments, partially purified R. typhi was incubated with affinity purified αRisk1 (20 μg) or αIgG isotype control (20 μg) Abs on ice for 30 min. Antibody-treated R. typhi were added to a monolayer of HeLa cells and incubated for various length of time at 34°C with 5% CO_2_. Samples were washed three times with 1× PBS and fixed with 4% PFA at RT for 20 min. To distinguish between extracellular and engulfed R. typhi, infected cells were stained first with αR. typhi Ab in 1× PBS containing 0.5% normal goat serum for 45 min at RT and then incubated with αAlexa Fluor 594 Ab in 1× PBS containing 0.5% normal goat serum for 30 min at RT. Next, cells were incubated for 10 min in permeabilization buffer (0.5% normal goat serum, 0.3% saponin in 1× PBS) and samples were reincubated with αR. typhi Ab in permeabilization buffer for 45 min at RT followed by incubation with an αAlexa Fluor 488 Ab in permeabilization buffer for 30 min. The cells were washed with 1× PBS and mounted with ProLong Gold antifade mounting medium containing DAPI. Images were acquired using the Nikon W-1 spinning disk confocal microcope (University of Maryland Baltimore, confocal core facility) and analyzed using Fiji software. Approximately 400 host cells were enumerated for each experiment and repeated in triplicates.

In experiments investigating the effects of Risk1 on the distribution of various cellular phosphoinositides, HeLa cells were transfected with 0.5 μg of pmCherry vector, pmCherry-myc-Risk1 WT, or pmCherry-myc-Risk1 H297A plasmid DNA, in combination with 0.5 μg of pGFP-PH_PLCδ_, pGFP-2xFYVE, or pGFP-PH_AKT_ plasmid DNA using PolyJet transfection reagent (Signagen) per the manufacturer’s recommendation. Changes in phosphoinositide distribution were monitored in cells that coexpressed the vector or Risk1 constructs with the corresponding phosphoinositide probes. Approximately 200 cotransfected cells were enumerated by confocal microcopy, and each experiment was repeated in triplicates. For assessing changes in cellular morphology, in particular, changes in area size between cells transfected with pGFP-vector, pGFP-Risk1 WT, or pGFP-Risk1 H297A were analyzed using the Coloc 2 plugin for Fiji software.

For experiments studying the association between endogenously expressed Beclin-1 or overexpressed Rab5 WT with Risk1, cells were transfected with 0.5 μg of pGFP-empty vector, pGFP-Risk1 WT, or pGFP-Risk1 H297A plasmid DNA alone or in combination with 0.5 μg of pCDNA3-myc-Rab5 WT DNA using the PolyJet reagent as described above. Twelve hours after transfection, cells were infected with partially purified R. typhi for various length of time as described above. Next, cells were lysed, immunoprecipitated, and analyzed by immunoblotting as described above.

### Flow cytometry.

For flow cytometry analysis, 1 × 10^6^ HeLa cells were transfected with 1 μg of pGFP-vector, pGFP-Risk1 WT, or pGFP-Risk1 H297A plasmid DNA using PolyJet. Twelve hours after transfections, cells were resuspended at a concentration of ∼0.2 × 10^6^ cells/0.1 ml in Cytofix/Cytoperm solution and incubated for 20 min on ice with 20 μl of fluorescein isothiocyanate (FITC)-conjugated anti-active-caspase-3 Ab (BD Pharmingen). Cells were washed 2 times with Perm/Wash buffer and finally resuspended in 200 μl of blocking buffer. Alternatively, transfected cells were stained with annexin V-FITC and 7-AAD using the annexin V-FITC apoptosis detection kit by following the manufacturer’s specifications (BD Pharmingen). For experiments utilizing the PI3K inhibitors, cells were transfected as described above and incubated for 6 h followed by an additional 6 h in the presence of wortmannin (1 to 5 μM), LY294002 (1 to 2 μM), or equal volumes of the diluent DMSO as a control. The percentage of apoptotic cells was assessed by flow cytometry of Risk1^+^ cells using BD Biosciences FACS Accuri C6 and FCS Express V6 software.

### Statistical analysis.

The statistical significance was assessed using analysis of variance with the Bonferroni’s posttest or by two-tailed unpaired Student's *t* tests (GraphPad Software, v. 6.0). Data are presented as means ± standard errors of the means (SEMs), unless stated otherwise. Alpha level was set to 0.05.
