# Supplementary material for: Risk1, a Phosphatidylinositol 3-Kinase Effector, Promotes Rickettsia typhi Intracellular Survival
Source: mBio. 2020 Jun 16;11(3):e00820-20. doi: 10.1128/mBio.00820-20 (PMC7298712; doi:10.1128/mBio.00820-20)
Supplement: FIG S2 [file mBio.00820-20-sf002.pdf]

FIG S2

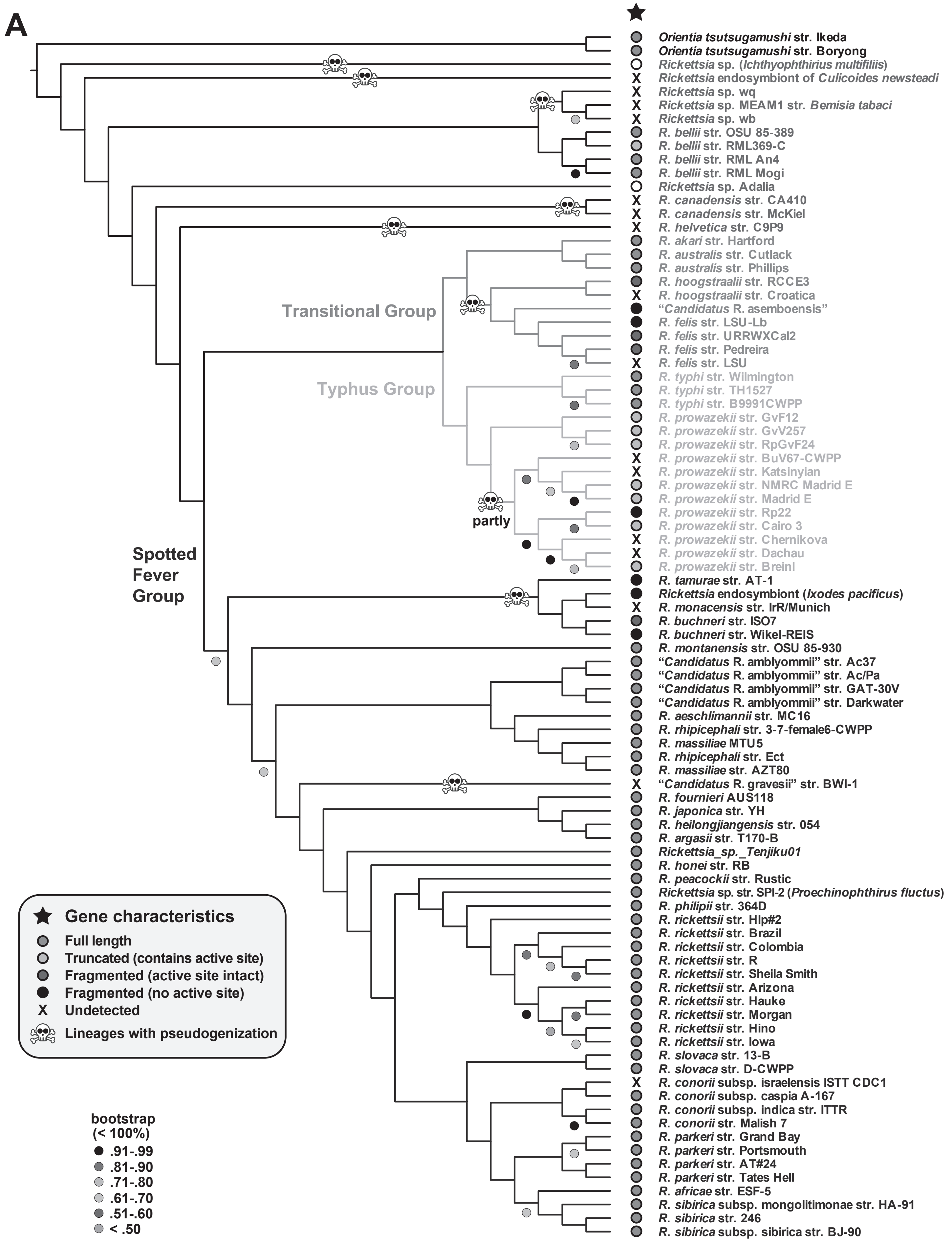
A
Orientia tsutsugamushi str. Ikeda
Orientia tsutsugamushi str. Boryong
Rickettsia sp. (Ichthyophthirius multifiliis)
Rickettsia endosymbiont of Culicoides newsteadi
Rickettsia sp. wq
Rickettsia sp. MEAM1 str. Bemisia tabaci
Rickettsia sp. wb
R. bellii str. OSU 85-389
R. bellii str. RML369-C
R. bellii str. RML An4
R. bellii str. RML Mogi
Rickettsia sp. Adalia
R. canadensis str. CA410
R. canadensis str. McKiel
R. helvetica str. C9P9
R. akari str. Hartford
R. australis str. Cutlack
R. australis str. Phillips
R. hoogstraalii str. RCCE3
R. hoogstraalii str. Croatica
"Candidatus R. asemboensis"
R. felis str. LSU-Lb
R. felis str. URRWXCal2
R. felis str. Pedreira
R. felis str. LSU
R. typhi str. Wilmington
R. typhi str. TH1527
R. typhi str. B9991CWPP
R. prowazekii str. GvF12
R. prowazekii str. GvV257
R. prowazekii str. RpGvF24
R. prowazekii str. BuV67-CWPP
R. prowazekii str. Katsinyian
R. prowazekii str. NMRC Madrid E
R. prowazekii str. Madrid E
R. prowazekii str. Rp22
R. prowazekii str. Cairo 3
R. prowazekii str. Chernikova
R. prowazekii str. Dachau
R. prowazekii str. Breinl
R. tamurae str. AT-1
Rickettsia endosymbiont (Ixodes pacificus)
R. monacensis str. IrR/Munich
R. buchneri str. ISO7
R. buchneri str. Wikel-REIS
R. montanensis str. OSU 85-930
"Candidatus R. amblyommii" str. Ac37
"Candidatus R. amblyommii" str. Ac/Pa
"Candidatus R. amblyommii" str. GAT-30V
"Candidatus R. amblyommii" str. Darkwater
R. aeschlimannii str. MC16
R. rhipicephali str. 3-7-female6-CWPP
R. massiliae MTU5
R. rhipicephali str. Ect
R. massiliae str. AZT80
"Candidatus R. gravesii" str. BWI-1
R. fournieri AUS118
R. japonica str. YH
R. heilongjiangensis str. 054
R. argasii str. T170-B
Rickettsia_sp._Tenjiku01
R. honei str. RB
R. peacockii str. Rustic
Rickettsia sp. str. SPI-2 (Proechinophthirus fluctus)
R. philipii str. 364D
R. rickettsii str. Hlp#2
R. rickettsii str. Brazil
R. rickettsii str. Colombia
R. rickettsii str. R
R. rickettsii str. Sheila Smith
R. rickettsii str. Arizona
R. rickettsii str. Hauke
R. rickettsii str. Morgan
R. rickettsii str. Hino
R. rickettsii str. Iowa
R. slovaca str. 13-B
R. slovaca str. D-CWPP
R. conorii subsp. israelensis ISTT CDC1
R. conorii subsp. caspia A-167
R. conorii subsp. indica str. ITTR
R. conorii str. Malish 7
R. parkeri str. Grand Bay
R. parkeri str. Portsmouth
R. parkeri str. AT#24
R. parkeri str. Tates Hell
R. africae str. ESF-5
R. sibirica subsp. mongolitimonae str. HA-91
R. sibirica str. 246
R. sibirica subsp. sibirica str. BJ-90
Transitional Group
Typhus Group
partly
Spotted Fever Group
Gene characteristics
Full length
Truncated (contains active site)
Fragmented (active site intact)
Fragmented (no active site)
X Undetected
Lineages with pseudogenization
bootstrap (< 100%)
.91-.99
.81-.90
.71-.80
.61-.70
.51-.60
< .50

FIG S2

B

```
O. tsutsugamushi  MKVLMYKKEITESILAKQLFESLALAGLSKTTDETILSLSQSLAKTLNSISKSFSTSSLEEYQKCF-------SNFNDHDLHKVLKVLKDTFAEDEYKEL   93
R. bellii         ---MAENETEQ---LSSKFFNILKDTDL--TKNANIQASSDNLANSIININKELNSETATAYLNEIEKINKEIPDFKEKKLAQMLTVIKDTLPSIQNPDV   92
R. akari          ---MYKEKTQQELLLQEQLFKALKTEGT--ELNAKISRACEAIASSAIDLSKDQSHTAASLYVNQI-----ALADLDGRGLAKVTTIIKDAFPGDKYRKF   90
R. typhi          ---MSKAKTKQELLLQQELFNVLKTHDI--EAHAKISRICERIAESVIALADQQSHTTESSYINYI-----NQAALNTKDLAKVTTTIKNFFPGDHNKKL   90
R. prowazekii     ---MSKEKITRELLLQQELFKALKTHDI--AANAKISRICRRIAGSVIALARQQSHTTENLYTHYI-----NQAAFNTKDLAKVTTIIKNLFPGDKNKKL   90
R. montanensis    ---MSKEKTQQELLLQEQLFKALKTNDT--ELNAKISRACEGIAESVIDLSNDQSPTAESLYISYM-----ALADLNTKDLAKVTTIIKNSFPGDKNKEL   90
R. aeschlimannii  ---MSKEKTQQELLLQEQLFKALKTNDT--ELNAKISRTCEGIAESVIDLSNDQSPTAESLYISYM-----ALADLNTKDLAKVTTIIKNSFPGDKNKEL   90
R. japonica       ---MSKEKTQQELLLQEQLFKALKTNDT--ELNAKISRTCEGIAESVIDLSNDQSPTTESLYISYM-----ALADLNTKDLAKVTTIIKNSFPGDKNKEL   90
R. rickettsii     ---MSKEKTQQELLLQEQLFKALKTNDT--ELNAKISRTCKGIAESVIDLSNDQSPTAESLYISYM-----ALADLNTKDLAKVTTIIKNSFPGDKNKKL   90
R. conorii        ---MSKEKTQQELLLQEQLFKALKTNDT--ELNAKISRTCKGIAESVIDLSNDQSPTAESLYISYM-----ALADLNTKDLAKVTTIIKNSFPGDKNKKL   90
                     :   :      * .::*: *   .        .*   .  :* :   :  . .  :    *    :         . :. . * :: . :*: :.      ..

O. tsutsugamushi  RNIIELPLLDRLTTKFKIERE------------KELSIQQSPNFNNSSIEELLRDKNVSKQSEFFQTGDKQDGVSAGYVANEKLSNNTFLLKKFYKNNDS  181
R. bellii         KEILDIPLIEYLAEKQSKE---------YNQEKENFE--KRGKVAPKAVSDLIRDRRVANVQEFKKEADKSEGVSGGYIADEQATHNTFMLKRFYKKGAE  181
R. akari          REIIDAPLLQHLVMEESIKRQVELENSRYNSDYKKFEEISNPNDPKKAIDAMLRDKKVAKQTEFEKKRKKAAGVSAGYVAQDE-LGNTFILKHFYRTHAA  189
R. typhi          REIIDTPLLQNLVIAGSIERKIGLNDTLYTPDYRKLEEIIDPNDTKKVIDAMLRDKGVAQQDEFEKKGKKTTGISAGYIANDS-AGNTFILKHFYKTHAA  189
R. prowazekii     REIIDTPLLQNLVIEGSIERKIELNDPQYNTNYRKFEEIIDPNETKKVIDAMLRDKRVAQQDEFEETGKKTAGISAGYVANDS-TGNTFILKHFYKTHAA  189
R. montanensis    REIIDAPLLQHLVMAEAIERQVGLDDSRYNADYRKREGIRNPNDPKKAIDAMLRDKRVAQQAEFEGKGKKAAGVSAGYVAKDN-AGNTFILKHFYKTHAA  189
R. aeschlimannii  REIIDAPLLQHLVMAEAIERQVGLDDSRYSADYRKLEGIRNPNDPKKAIDAMLRDKRVAQQAEFEGKGKKAAGVSAGYVAKDN-AGNTFILKHFYKTHAA  189
R. japonica       REIIDAPLLQHLVMKEAIERQVGLDDSQYNADYRKLEEIRNPNDPKKVIDAMLRDKRVAQQAEFQGKGKKTAGVSAGYVAKDN-AGNTFILKHFYKTHAA  189
R. rickettsii     REIIDTPLLEHLVMIEAIERQVGLDDSRYNADYRKLEEIRNPNDPKKVIDAMLRDKRVAQQAEFEEKGQKAAGVSAGYVAKDN-AGNTFILKHFYKTHAA  189
R. conorii        REIIDAPLLEHLVMIEAIERQVGLDDSRYNADYRKLEEIRNPNDPKKVIDAMLRDKRVAQQAEFEEKGKKAAGVSAGYVAKDN-AGNTFILKHFYKTHAA  189
                  .:*:: **:: *.      :                 : .      :    . :. ::**. *::  **     .*   *:*.**:*.:.   ***:**.**..

O. tsutsugamushi  VLAIMDPAERQKAQNDVRDAVQELLTSSMYKLLLYGRAPKEELVT--PDEVNSD----LLYIRSKFFENAVLLSEFSGSKNSIHIKADAKNLQELEGFEK  275
R. bellii         VETTQQWM-------DRNDAVRELIGGNMYEQLLYNRAPKEELVTGYANSKLPDFVSKILYVRSKFLENSEHLGEI----------LKETNVPKLAGFEK  264
R. akari          CEKIQDNNARQQAIDDRRDGMQELIGSTMYQFLLHDRAPKEGLVK--ADEKHPD----ALYVRSKFFDNAVTLTEFSGLSDHTKVRSDDQNLKKLKGFEK  283
R. typhi          CEKIQDPIEQSHAIDDRRDGVQELIGSTMYQFLLHDRAPKEGLVK--ADAQHPD----SLYVRSKFFDNAVTLTEFCGLSGQTRVRVNDKNLKKLEGFEK  283
R. prowazekii     CKKIQDPKEQDQAITDRRDGVQELIGSTMYQFLLHDRAPKEGLVN--VDEQHPD----SLYVRSKFFDNAVTLAEFCGLSGLTRVRINDKNLKRLEGFEK  283
R. montanensis    CQKIQDNKAQKQAIADRRDGVQELIGSTMYQFLLHDRAPKEGLVT--ADEKHPD----SLYVRSKFFDNAVTLTEFSGLSGQTRVRANDQNLKKLEGFEK  283
R. aeschlimannii  CQKIQDNKAQKQAIADRRDGVQELIGSTMYQFLLHDRAPKEGLVT--ADEKHPD----SLYVRSKFFDNAVTLTEFSGLSGQTRVRANDQNLKKLEGFEK  283
R. japonica       CKKIQDIQAQRQAVADRRDGVQELIGSTMYQFLLHDRAPKEGLVT--ADEQYPD----SLYVRSKFFDNAVTLTEFSGLSGETRVRANDQNLKKLEGFEK  283
R. rickettsii     CQKIPDNNAQKQAMADRRDGVQELIGSTMYQFLLHDRAPKEGLVT--ADEKHPD----SLYVRSKFFDNAVTLTEFSGLSGETRVRANDQNLKKLEGFEK  283
R. conorii        CQKIQGNHAQRQAMADRRDGVQELIGSTMYQFLLHDRAPKEGLVT--ADEQHPD----SLYVRSKFFDNAVTLTEFSGLSGETRVRDNDQNLKKLEGFEK  283
                               * .*.:.**: ..**: **:.***** **.    :    .*      **:****::*:  * *:           .  *:  * ****

                          Catalytic loop    Activation loop
O. tsutsugamushi  IIASCHILGEADYHPGNLMVQDGKTVVKIDHGRSMMQFHKDFESMVKSTYNIFNHNQIGYNSAIQAGNLSFNIEKYNAALNQMLTQLDEYQIDTVVEKKA  375
R. bellii         AIAASHILGESDYHTQNLMVQNNNTVVKIDHGRSFMNFNKDFTSMLQDANEKFER--FGYNENIEKGQLTFNVKEYSKSLNQMLNQLDNNQIDNIIDQKV  362
R. akari          AIAACHMLGEADYHAGNLMVQDGKTITKIDHGRSFLAFHKDFGSMTQSTNRMFSHPGVGYSAAIKKGNLSFSIDKYSESLNQMISQFDEQQMAAIVDQKI  383
R. typhi          AIAACHMLGDGDYHAGNLMVQNGKTITKIDHGRSFLEFHQDFGSMVQLTAEMFTHPNVCYSDAIKAGNLSFNIDKYSEALNQMISQFDVQRMEAIVDQKL  383
R. prowazekii     AIAACHILGDGDYHAGNLMVQNGKTITKIDHGRSFLEFHKDFASMIQSTAEMFIHPHVCYSYAIKAGNLSFNIDKYSEALNQMINQFDLKHMEAIVDQKL  383
R. montanensis    AIAACHMLGEVDYHAGNLMVQDGKTITKIDHGRSFLAFHKNFGSMIQSTAKMFAHPGVGYSAAIKAGNFSFSINKYSESLNQMISQFDEKQMEAIVDQKI  383
R. aeschlimannii  AIAACHMLGEVDYHAGNLMVQDGKTITKIDHGRSFLAFHKNFGSMIQSTAKMFAHPGVGYSAAIKAGNFSFSIDKYSESLNQMISQFDEKQMEAIVDQKI  383
R. japonica       AIAACHMLGEVDYHAGNLMVQDGKTITKIDHGRSFLAFHKNFGSMIQSTAKMFTHPGVGYSAAIKAGNFSFNIDKYSESLNQMISQFDEKQMEAIVDQKI  383
R. rickettsii     AIAACHMLGEVDYHAGNLMVQDGKTITKIDHGRSFLAFHKNFSSIIQSTAAMFTHPGVGYSAAIKAGNFSFSIDKYSASLNQMISQFDEKQMEAIVDQKI  383
R. conorii        AIAACHMLGEVDYHAGNLMVQDGKTITKIDHGRSFLAFHKNFGSMIQSTAEMFTHPGVGYSAAIKAGNLSFSIDKYSESLNQMISQFDEKHMEAIVDQKI  383
                   **:.*:**: ***. *****:.:*:.*******:: *:::* *: : :    *  .  . *.   *: *:::*.:.:*. :****:.*:*  .:   ::::*

O. tsutsugamushi  SELQKAGFNPKGLMAKVRFDDETFMHTTISNYQDFKAFYKDNVKENLQNMRRIAQTTDIISKFSNVSPEFKNGRWLKDFAISDIKEPVIYASFHNIEIEG  475
R. bellii         DELVKNGFDAKGICPGKLDIKGLDTSVIFNNEQELRKYYKDNIKENLSNMKEIAKKVDIVAKYSNPSPDFENGKWLKDMANSPMQDPVAYAAHNDIQIEG  462
R. akari          DELKQAGFDPKNVA----------LPTKINNFDDLRQYYKSSIKANLINMQEIAKGAEIVTKFTNVSPKFNNGGWLEAFAHSPVKDPVLYAIYNNIKIEG  473
R. typhi          DELKKAGFDPDNIP----------LYININNFDDLKQYYKSSIKQNFINMQEIAKGAEIVKKFSNVLPEFKNGGWLEAFANSPVKEPLLYAIKNKITIEG  473
R. prowazekii     DELKKAGFDPNKIP----------LSINIKNFDDLKKYYKTHIKQNIINMQEVAKGAEIVKKFSNVLPSFKNGGWLEAFANSPIKEPLLYAIENNITIEG  473
R. montanensis    DELKKAGFDPKNIA----------LSTNIKNFDDLRKHYKGSIKENLVNMQEVAKGAEIVTKFSNVLPEFNNGGWLEAFANSPVKDPVLYAIDNNITIED  473
R. aeschlimannii  DELKKAGFDPKNIA----------LSTNIKNFDDLRKHYKGSIKENLVNMQEVAKGAEIVTKFSNVSPEFKNGGWLETFANSPVKDPVLYAIDNNITIEG  473
R. japonica       DELKKAGFDPKNIA----------LSTNIQNFDDLRKHYKGSIKEHLVNMQEVAKGAEIVTKFSNVLPEFKNGGWLEAFANSPVKDPVLYAIDNNITIEG  473
R. rickettsii     DELKKAGFDPKNIM----------LSTNIQNFDDLRKHYKSSIKENLVNMQEVAKGAEIVTKFSNVSPEFKNGGWLEAFAHSPVKDPVLYAIDNNITIEC  473
R. conorii        DELKKAGFDPKNIM----------LSTNIQNFDDLRKHYKSSIKENLVNMQEVAKGAEIVTKFSNVSPEFKNGGWLEAFANSPVKDPVLYAIDNNITIEG  473
                  .** : **:.. :                 :.* :::. .**  :* :: **. :*: .:*: *::*  *.*:** **: :* * :::*: **  :.* **

O. tsutsugamushi  KSALEWAISNKYHAVENV---------------------------IKAEKVLKGIEDELFPLVQRLQ---------------------------------  515
R. bellii         KNALQWAHENNYKIEIPTSEVKTEKVTENQWSKNANGKWEETPIEVEKKQKEYRSIDPLKYILSRNQVAISKSEKEFLNQAINLGIKNNFDESYKENTRF  562
R. akari          INAKDWAYEHNYQIHTYIG-LKQAVVKEQQWCKDLEGKWTEKEVEVQKDTVEVKITDPVKSM--REQ---------------------------------  537
R. typhi          KDAQEWAYENNYQIKTAIG-LTKEVIKERQWRKDFEGKWQEKEIEVQRDKVEVQLSDPLKSM--RIQ---------------------------------  537
R. prowazekii     KDAQEWAYENNYQIKTAIG-LTKETIKEQQWSKDFEGKWQEREVEIQRDKVEIQLSDPLKSI--RIQ---------------------------------  537
R. montanensis    KDAKEWAYENNYQIKISIG-LKKDIIKEQQWNKTLDGKWKEKEVEVKTDKVEVQISDPAKSM--RIQ---------------------------------  537
R. aeschlimannii  KDAKEWAYENNYQIKISIG-LKKEIIKEQQWSKDLDGKWKEKEVEVKTGKVEVKITDPAKSM--RIQ---------------------------------  537
R. japonica       KDAKEWAYENNYQIKISIG-LKKEIIREQQWSKDLDGKWKEKEVEVKTDKVEVHIADPAKSM--RIQ---------------------------------  537
R. rickettsii     KDAKEWAYENNYQIKIAIG-LKKETIKEQQWSKDLDGKWKEKEVEVKTDKVEVQIADPAKSM--RIQ---------------------------------  537
R. conorii        KDAKEWAYENNYQIKISIG-LKKETIKEQQWSKDLDGKWKEKEVEVKTDKVEVQISDPAKSM--RIQ---------------------------------  537
                   .* :** .::*:                                ::          *     :   * *
```

FIG S2

B (cont'd.)

```
O. tsutsugamushi  ------------D-----------------TPMKKLTLKKVKQFYDSVLTSFEQKQYLTQQEVKDIQECLDYKKHIENTTAFIKLNI-ESISMVDKMRYK  585
R. bellii         KDNSIVKNLNKEDKTITNEIEKLAQKFVKNNSKNTITTEAMEKLYDDTLQIMKDKKLLTDQDIATIKNDKRFKERIKNTTEFINNENKFDISRTDKVYYK  682
R. akari          ------------DKSVGAKLESLIVAFTKQAITQHVTDKKVTKFYDNIMKTLKKEKYLTEQDIEGIKKDLKYWDNIENTTNLLNAKS-FKLNVKDTTYYK  624
R. typhi          ------------DKFTAKKFESLIINFTKHTTTKNVTDEEVEKFYDNIMQVLKKENYLNEQDIETIKQDLKYHDNIENTTHLLNAKD-LKLNSKDTIYYK  624
R. prowazekii     ------------DKFTAEKLGSLIVNFTKHTTTKNVTDKEVAKFYDNIMKVLKK-------------------------SIILPSKM-LKL.........  590
R. montanensis    ------------DESTGEKLASLIVDFTKQATTQNVTDKAVAKFYDNIMKALKKENYLTEQDIEGIKKNLKYQDNIENTTNLLNAKS-FKLNSKDTIYYK  624
R. aeschlimannii  ------------DKSTGEKLASLIVDFTKQTTTKNVTDKAVAKFYDNIMKVLKKENYLTEQDIEGIKKNLKYKDNIENTTNLLNAKS-FKLTAKDTIYYK  624
R. japonica       ------------DKSTGEKLASLIVDFTKQATTKNVTDKAVAKFYDKIMKVLKKENYLTEQDIKGIKKNLKYQDNIENTTNLLNAKS-FKLNSKDTIYYK  624
R. rickettsii     ------------DKSTGEKLASLIVDFTKQATTKNVTDKAVAKFYNNIMKVLKKENYLTEQDIKGIKKNLKYQDNIENTTNLLNAKS-FKLNYKDTIYYK  624
R. conorii        ------------DKSTGEKLASLIVDFTKQATTKNVTDKAVAKFYDNIMKVLKKENYLTEQDIKGIKKNLKYQDNIENTTNLLNAKS-FKLNSKDTIYYK  624
                              *                    : :* : : ::*:. :  ::.                         : ::  :   .:

O. tsutsugamushi  LATFCNILSLPSKAKYFMDRITPESLNIIQQLKKDISNSSGFKKVLLKS--------SPNYK--SNTHSTYSISKSKNDSNNQKGILM  663
R. bellii         LARFYKKIGFKGIANDLKAKIDPETLNKINKFENVQNITSDIREVLMNNRSGKEGIQTARLNKMNIKLDIQPKLNTKPKNRKQESTQR  750
R. akari          VGNFCEKIGLSNIANYCMRQISPNNLSKIHDTQKLIAASIKIGNILQEKG--NRGIKPKRVE--AIKVIAFSQLQAKSENHRQR----  704
R. typhi          IGNFCEKIGLRSVANHFIKKIAPENLNKIYNTEKVIAESIKIGNILKDNK--QEGLQTKRLE--TVKGIVVSQLQAKQGKQQQGKHY-  707
R. prowazekii     ........................................................................................  590
R. montanensis    VGNFCEKRGLPSISNYFIKQIAPENLNKIHNTEKLIAESIKIGNVLQQNK--QAGLQTKRVE--TVKGIAFSKLQARKEIHQQR----  704
R. aeschlimannii  VGNFCENRGLPSISNYFMKQISPENLNKIHNTEKLIAESIKIGNVLQQKK--QEGLQTKRVE--TVKGIAFSKLQARKERHQQR----  704
R. japonica       VGIFCEKIGLSSISNYFMKQISPENLNKIHNTEKLITESIKIGNVLQQKK--QKGLQTKRVE--TVKEIACSKLQARKERHQQR----  704
R. rickettsii     VGIFCEKRGLPSISNYFMKQISPENLNKIHNTEKLIAESIKIGNVLQQKK--QERLQTKRVE--TVKEIAFSQLQARKERHQQR----  704
R. conorii        VGIFCEKRGLPSISNYFMKQISPENLNKIHNTEKLIAETIKIGNILQQKK--QERLQTKRVE--TVKEIAFSQLQARKERHQQR----  704
```
